# Intraluminal neutrophils limit epithelium damage by reducing pathogen assault on intestinal epithelial cells during *Salmonella* gut infection

**DOI:** 10.1371/journal.ppat.1011235

**Published:** 2023-06-29

**Authors:** Ersin Gül, Ursina Enz, Luca Maurer, Andrew Abi Younes, Stefan A. Fattinger, Bidong D. Nguyen, Annika Hausmann, Markus Furter, Manja Barthel, Mikael E. Sellin, Wolf-Dietrich Hardt

**Affiliations:** 1 Institute of Microbiology, Department of Biology, ETH Zurich, Zurich, Switzerland; 2 Science for Life Laboratory, Department of Medical Biochemistry and Microbiology, Uppsala University, Uppsala, Sweden; 3 reNEW – NNF Center for Stem Cell Medicine, University of Copenhagen, Denmark; University of California Davis School of Medicine, UNITED STATES

## Abstract

Recruitment of neutrophils into and across the gut mucosa is a cardinal feature of intestinal inflammation in response to enteric infections. Previous work using the model pathogen *Salmonella enterica* serovar Typhimurium (*S*.Tm) established that invasion of intestinal epithelial cells by *S*.Tm leads to recruitment of neutrophils into the gut lumen, where they can reduce pathogen loads transiently. Notably, a fraction of the pathogen population can survive this defense, re-grow to high density, and continue triggering enteropathy. However, the functions of intraluminal neutrophils in the defense against enteric pathogens and their effects on preventing or aggravating epithelial damage are still not fully understood. Here, we address this question via neutrophil depletion in different mouse models of *Salmonella* colitis, which differ in their degree of enteropathy. In an antibiotic pretreated mouse model, neutrophil depletion by an anti-Ly6G antibody exacerbated epithelial damage. This could be linked to compromised neutrophil-mediated elimination and reduced physical blocking of the gut-luminal *S*.Tm population, such that the pathogen density remained high near the epithelial surface throughout the infection. Control infections with a *ssaV* mutant and gentamicin-mediated elimination of gut-luminal pathogens further supported that neutrophils are protecting the luminal surface of the gut epithelium. Neutrophil depletion in germ-free and gnotobiotic mice hinted that the microbiota can modulate the infection kinetics and ameliorate epithelium-disruptive enteropathy even in the absence of neutrophil-protection. Together, our data indicate that the well-known protective effect of the microbiota is augmented by intraluminal neutrophils. After antibiotic-mediated microbiota disruption, neutrophils are central for maintaining epithelial barrier integrity during acute *Salmonella*-induced gut inflammation, by limiting the sustained pathogen assault on the epithelium in a critical window of the infection.

## Author summary

Microbiota and mucosal immune system work in coordination to protect our intestine against enteric infections under homeostasis. Dysregulation of this defense increases the risk of inflammatory diseases. Infectious agents such as *Salmonella enterica* or *Clostridioides difficile* trigger particular neutrophil responses, namely crypt abscesses or pseudomembranous colitis. These are characterized by pronounced neutrophil influx and aggregate formation in the gut lumen. However, if these neutrophils are protective or damage-inducing is not well understood. Here we use mouse models for *Salmonella* Typhimurium to study this question. We observed that luminal neutrophils constitute an important part of the coordinated immune response to *Salmonella* invasion of the gut epithelium. In the absence of neutrophils, the continuous pathogen attacks on the epithelial cells overwhelm the system and lead to elevated epithelial cell loss. We show that intraluminal defense is especially important if the microbiota is perturbed or missing. Infection of germ-free mice lacking neutrophil defense leads to the complete destruction of the epithelial layer. This work provides new insights into the protective role of microbiota and intraluminal neutrophils, and how they together ensure the maintenance of epithelial barrier integrity against enteric infections.

**Highlights**

○ After the first wave of mucosal invasion (day 1 p.i.), *S*.Tm maintains the assault from the lumen, triggering the continued expulsion of epithelial cells in antibiotic pretreated mice.
○ Neutrophil recruitment into the gut lumen is essential to limit this continued *Salmonella* attack on the epithelium.
○ In antibiotic pretreated SPF mice, neutrophil depletion exacerbates *S*.Tm invasion, causing excessive epithelial cell loss, which compromises epithelial barrier integrity at later time points (day 2–3 p.i.).
○ In germ-free mice, neutrophil depletion exacerbates epithelial responses and epithelial barrier destruction even more potently than in streptomycin pretreated SPF mice.
○ Gentamicin treatment and *ssaV* mutant infections indicate that neutrophils prevent epithelial damage by eliminating and physically blocking gut-luminal pathogens.

## Introduction

Polymorphonuclear leukocytes (PMN), mainly compromised of neutrophils, are the most abundant immune cell type in circulation and considered as an early line of defense against many infections, including those caused by enteropathogenic bacteria. Neutrophil recruitment into and across the gut epithelium is a hallmark of infectious (e.g., pathogen-induced) and non-infectious (e.g., inflammatory bowel disease) enterocolitis [1–6]. They provide a powerful defense during enteric infections using a myriad of effector mechanisms (i.e., reactive oxygen/nitrate species (ROS/RNS), antimicrobial peptides (myeloperoxidase/neutrophil elastase; MPO/NE), and neutrophil extracellular traps (NETs) [7,8]. While being indispensable in the defense against microbes, neutrophils are also often associated with tissue damage during gut inflammation [5]. Therefore, the degree of protective versus tissue damaging effects of neutrophils is highly context dependent and is not fully understood for all natural infections and infection models traditionally used in the field.

Under homeostasis, the gut microbiota provide efficient colonization resistance and thereby prevent enteropathogen infections and subsequent enteropathy.[9] Compromising colonization resistance increases the risk of enteric disease.[9] Intestinal inflammation caused by enteric pathogens such as non-typhoidal *Salmonella enterica* serovars (NTS), including serovar Typhimurium (*S*.Tm) has been extensively studied in recent years. In *S*.Tm infection models, the microbiota is often perturbed to permit efficient infections via the orogastric route. Murine models of *S*.Tm gut inflammation such as the streptomycin pretreatment of C57BL/6 mice provide an excellent basis to study the stages of gut inflammation during *Salmonella* infection [10,11]. To elicit disease in streptomycin pretreated mice, *S*.Tm invades intestinal epithelial cells using its type-three secretion systems (TTSS)-1 and -2 [2]. Invasion of the epithelial cells activates the NLRC4 inflammasome. This results in two major outcomes: i) expulsion of infected epithelial cells into the gut lumen [12] and ii) recruitment of innate immune cells including neutrophils via secretion of IL-18 and other immune mediators [13,14]. Studies in this model suggest that neutrophils may have stage-specific functions during the acute infection. In the first 18h after an oral dose of *S*.Tm, neutrophils appear not to be involved in defense against *Salmonella*, at least in C57BL/6 mice [13]. Follow-up research in mice infected for up to 4 days indicated that neutrophils may impose a strong bottleneck, reducing the gut luminal *S*.Tm population by day 2 p.i. to as little as 15,000 *S*.Tm cells [14]. However, in that study neutrophils were depleted in C57BL/6 mice with an anti-Gr-1 antibodies (which depletes neutrophils and monocytes) [14]. Therefore, the true role of neutrophils versus monocytes in restricting the gut-luminal *S*.Tm population remains unclear in C57BL/6 mice. Regardless, the pathogen population can grow back to approx. 10^9^ CFU / g feces (by 96h p.i.) after passing the bottleneck. It remains unclear if these high pathogen densities in the gut lumen may represent an important continued driver of enteropathy at that stage of the infection as well. Similarly, due to their stage-specific effects, it remains unclear whether the recruitment of neutrophils is beneficial or rather a detriment for gut mucosal integrity during acute *Salmonella* infection.

Infected epithelial cells are expelled during enteric diseases to prevent the subsequent transmigration of the pathogens into the underlying tissue (lamina propria; LP) and the systemic organs [12,15–18]. Moreover, the expulsion may limit the amounts of inflammasome-dependent cytokines like IL-18 which are released into the lamina propria. The resulting immune responses lead to temporary shortening of the intestinal crypts and require the rapid division of crypt stem cells to retain barrier integrity (crypt hyperplasia; [11,16,19]). A failure to replace the expelled epithelial cells, as reported in germ-free mice infected with wild type *S*.Tm [19], can lead to shortened crypt structures and can be detrimental to epithelial barrier integrity. Similarly, excessive assault on epithelial cells and increased pathogen tissue loads due to the lack of one or more of the mucosal defenses can also drive pathological cell loss and destruction of the epithelium. This was shown in the examples of NAIP/NLRC4 or GSDMD (downstream of NLRC4 inflammasome; executer of pyroptotic cell death [20]) deficiency, where failure to initiate timely epithelial immune responses resulted in increased pathogen loads and disruption of the epithelium at later stages of the infection [21,22]. These reports highlight the importance of a balanced and timely mucosal immune response to maintain epithelium integrity during enteric infections. However, as wild type *S*.Tm colonizes the gut lumen, the gut epithelium, and the lamina propria, it remained unclear which of these pathogen populations would be targeted by the neutrophil defense and how this affects the epithelial barrier. We hypothesized that the additional layer of defense which is conferred by neutrophils in the gut lumen might be crucial for easing the burden on the gut epithelium and thereby promote epithelial integrity during severe stages of acute *Salmonella* infection.

Here we used a comprehensive approach to assess the function of intraluminal neutrophils during *Salmonella*-induced gut inflammation. Utilization of mouse models with varying severity of *S*.Tm-induced colitis allowed us to highlight the stages of gut inflammation where neutrophils exert a crucial epithelium-protective function in the infected gut.

## Results

### Neutrophils slow down the progression of cecal tissue infection by day 3 of *S*.Tm infection

We have applied a cell depletion strategy to assess the role of neutrophils in controlling disease progression between days 1 to 3 in streptomycin pretreated C57BL/6 mice. Earlier work had tested the role of neutrophils in gut luminal bottlenecks by α-Gr-1 depletion in C57BL/6 mice, or by α-Ly6G depletion in 129 mice [14]. The effect of α-Ly6G depletion in C57BL/6 mice remained untested. Also, it remained enigmatic whether the tissue-lodged or the re-growing luminal pathogen population contribute to the enteropathy by day 3. Since we wanted to perform our study of neutrophils in the widely used C57BL/6 mouse background, we started out by depleting neutrophils in C57BL/6 mice using α-Ly6G, which is highly specific for neutrophils, but spares monocytes [23]. To this end, we orally infected streptomycin pretreated C57BL/6 mice with 5x10^7^ CFU of wild-type *S*.Tm (SL1344) for 2 or 3 days, as indicated. To relate our new data to the previous work, to detect possible bottlenecks on the gut luminal pathogen population, and to probe the contribution of neutrophils, we included seven wild-type isogenic tagged *S*.Tm strains in the inoculum (WITS; [24,25]) at a ratio of 1:1000 (tagged: untagged). This ratio of tagged versus untagged wild-type strains will result in the random loss of abundance in one or more of the tagged strains if the gut-luminal *S*.Tm population undergoes a transient bottleneck. Importantly, the resulting unequal tag-distribution will be maintained within the luminal *S*.Tm population, even after the population has passed through the bottleneck and has re-grown to carrying capacity at day 3 p.i.. Therefore, qPCR analysis of the tag abundances allows us to quantitatively assess gut luminal bottlenecks in the streptomycin pretreated mouse model [14].

**Fig 1:**
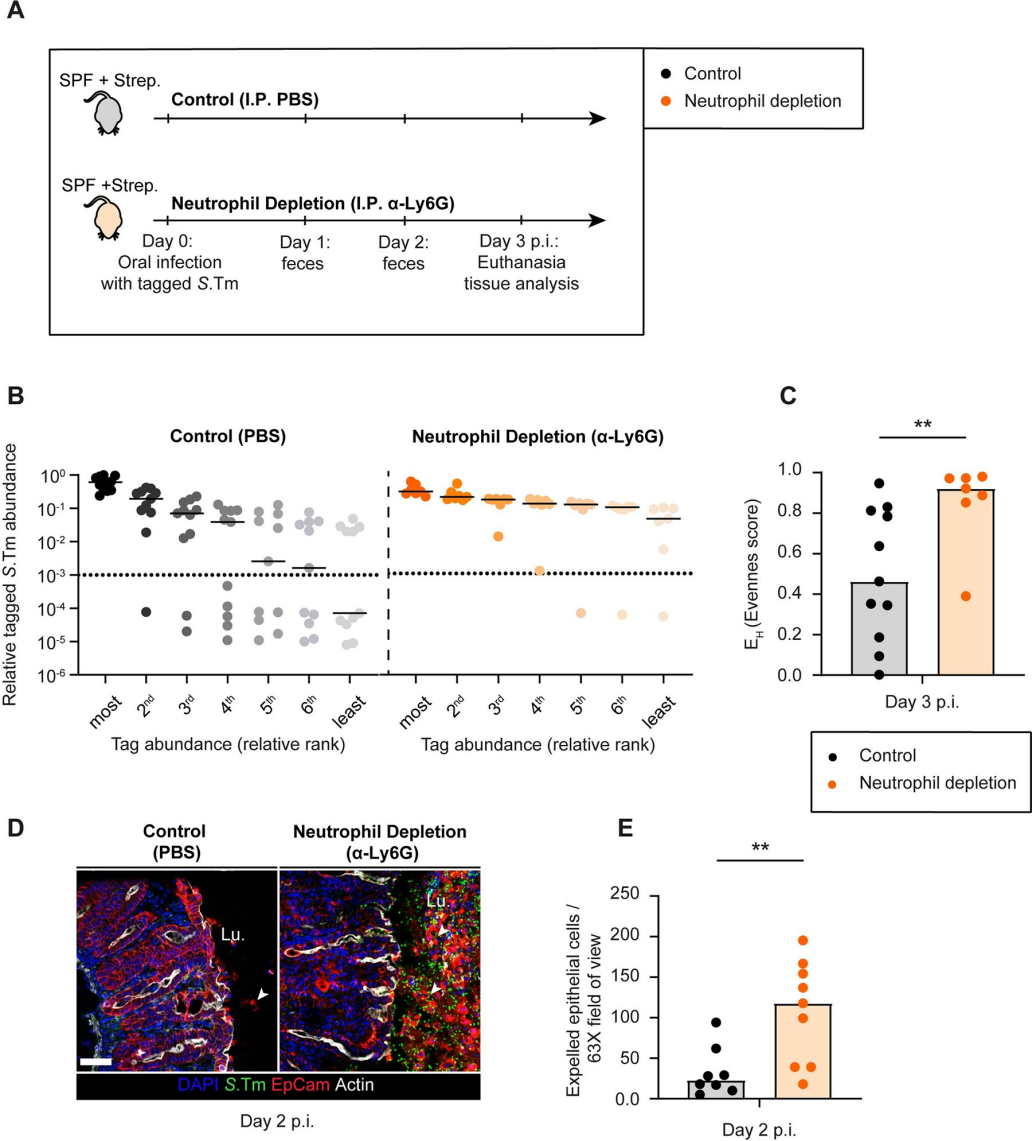
Consequences of neutrophil depletion with α -Ly6G on luminal pathogen loads and epithelial cell expulsion. **A)** Scheme summarizing the experimental setup of Panels **B-E**. Streptomycin pretreated C57BL/6 mice were infected orally with 5x10^7^ CFU of wt *S*.Tm (SL1344) for 3 days. One group (control) treated with the vector (PBS; black symbols) and the second group with α-Ly6G (orange symbols) intraperitoneally (I.P.). **B)** Relative ranks of the tagged *S*.Tm strain abundances in feces at day 3 p.i.. **C)** Evenness score at day 3 p.i. **D)** Representative micrographs of cecal tissue sections, stained for epithelial marker EpCam and *Salmonella* LPS. Lu. = Lumen. White arrows point at expelled epithelial cells. Scale bar = 50 μm. **E**) Microscopy-based quantification of expelled IECs per 63x field of view (i.e., cells/high power field; hpf). Each data point is the average of 5 fields of view (FOV) per section. Lines or Upper ends of the bars indicate the median. Dotted lines indicate the detection limit. **Panels B-C**) Pooled from 2 independent experiments for each group: control (n = 11 mice) and neutrophil depletion (n = 7 mice). **Panels D-E**) Pooled from 3 independent experiments for each group: control (n = 8 mice) and neutrophil depletion (n = 9 mice). Two-tailed Mann Whitney-U tests were used to compare two groups in each panel. p<0.01 (**).

To assess the role of neutrophils specifically, we treated one group with α-Ly6G (cl. 1A8; via intraperitoneal injection (I.P.); see details in **Materials & Methods**). This approach reduces the number of neutrophils recruited to the gut tissue during *Salmonella* infection (**Fig 1A**; [13]). Comparison to mock-depleted mice established that the anti-Ly6G depletion had no effect on the total fecal pathogen densities and promoted systemic pathogen spread by up to 10-fold by day 3 (**S1A, S1B and S1G Fig**). Strikingly, the α-Ly6G treatment ameliorated the gut-luminal bottleneck, as indicated by the higher evenness of the WITS-tagged strain distribution in the neutrophil depleted mice by day 3 p.i. (**Fig 1B and 1C** and **S1C and S1D Fig**). 24 out of 77 WITS were lost in the control (evenness score: ≈0.46; **Fig 1B and 1C**), while neutrophil-depleted mice retained a much higher fraction of all tags (only 3 out of 49 WITS were lost with an evenness score: ≈0.92; **Fig 1BC and 1C**). These data recapitulated the phenotypes observed in anti-Gr-1 depleted C57BL/6 mice [14] and indicated that this bottleneck is specifically attributable to neutrophils, but not the monocytes which had likely been depleted together with the neutrophils in that earlier study.

Of note, the high variability in the evenness score of control mice (**Fig 1B and 1C**, left side) can be attributed to the stochastic nature of our approach, in which one or more tags are randomly lost due to neutrophil-dependent killing. It is also interesting to note that the luminal pathogen densities in the cecal content of the α-Ly6G depleted mice were significantly higher than in the control mice both at day 2 and at 3 p.i. (**S1E Fig**). This was again in line with the earlier data from anti-Gr-1 depleted mice [14] and indicated that neutrophils can reduce the gut-luminal pathogen population, at least to some extent.

Importantly, these experiments provided us with materials to study the effects of the neutrophil depletion on the damage to the gut epithelium, a phenotype which had not been assessed in the earlier work.

To assess the degree of enteropathy, we focused on the cecal tissue, as this is the main site of invasion in the streptomycin pretreated mouse model [11]. Strikingly, fluorescence microscopy of cecal tissue at day 2 p.i. revealed massive epithelial shedding in α-Ly6G depleted mice, while the control mice showed much fewer epithelial cell expulsion events (**Fig 1D and 1E**). Of note, in the streptomycin mouse model, the degree of epithelial cell expulsion may differ between different mice as many parameters can affect the total outcome due to site-to-site variability in the cecum tissue sections of the same mouse monitored for epithelial cell expulsion, mouse-to-mouse variability in the efficiency of the neutrophil depletion, and slight mouse-to-mouse variations in the time course of disease progression.[26]. Nevertheless, the mice with neutrophil depletion had on average 5-fold more expelled epithelial cells in the cecum lumen and showed a disrupted crypt structure, whereas the control mice featured the typical infection-associated crypt hyperplasia by day 2 p.i. (**Fig 1D**). At this stage, epithelial cells often dislodged from the mucosa in the form of large cell aggregates, which is different from the typical NAIP/NLRC4-driven epithelial cell expulsion at 12-18h p.i. that we and others described before, where single infected cells are selectively extruded by their neighbours [12,22,27]. However, the underlying causes remained unclear.

We reasoned that in the absence of neutrophils, epithelial cells might be subject to more *S*.Tm invasion events as the gut luminal pathogen loads remained higher than in the non-depleted controls during the bottleneck-phase of the infection (i.e. day 2 p.i.). This might result in higher net pathogen invasion into the gut tissue. To test this, we quantified the pathogen loads in the cecal tissue using a gentamicin protection assay. We found elevated pathogen densities in the cecal tissue (and systemic sites) of neutrophil-depleted mice compared to control mice at day 3 p.i. (**S1F and S1G Fig**). We reasoned that higher tissue loads might result from a higher frequency of pathogen attacks from the luminal side in neutrophil depleted mice. Taken together, these observations suggest that in the absence of neutrophil defense, the gut mucosa experiences more *S*.Tm invasion attempts at ≈2–3 days of infection, coupled to excessive and uncontrolled shedding of intestinal epithelial cells into the lumen.

### Neutrophils provide a physical barrier against *S*.Tm invading from the gut lumen

**Fig 2:**
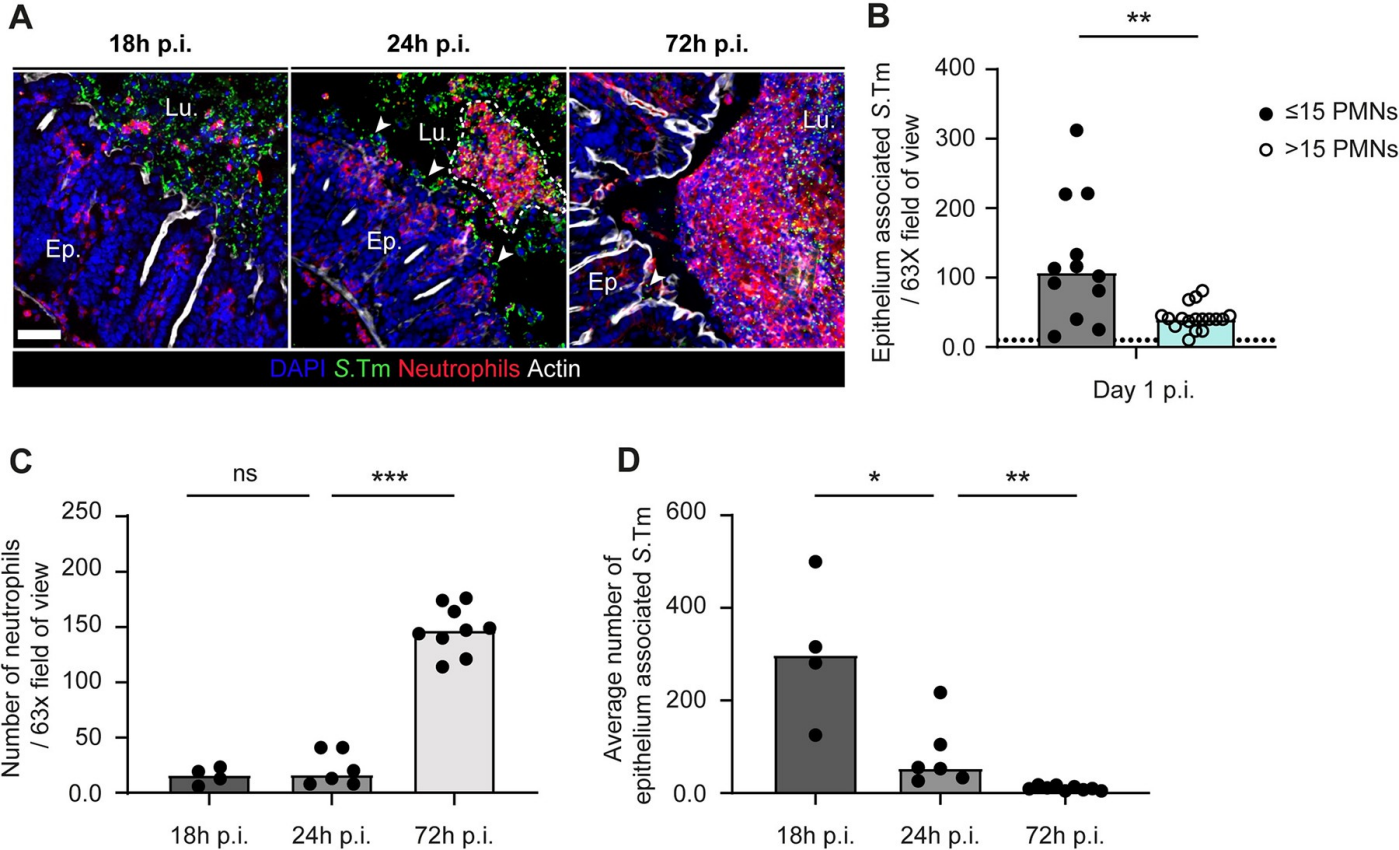
Microscopy analysis of the time course of neutrophil infiltration into the gut lumen (18h p.i., 24h p.i., and 72h p.i.). **A)** Representative micrographs of cecal tissue sections from mice infected with *S*.Tm for 18h, for 1 day (24h), or for 3 days stained for neutrophil marker Ly6B.2 and *Salmonella* LPS. Lu. = Lumen. Ep. = Epithelium. White arrows indicate *S*.Tm associated with the epithelium. Scale bar = 50 μm. **B-D)** Microscopy-based quantification of **B)** *S*.Tm associated with the epithelium at day 1 p.i.. (total 24 FOVs from 6 mice; filled symbols; neutrophils per field≤15 vs empty symbols; neutrophils per field>15). **C)** neutrophils per 63x field of view (each data point is the average of 5 FOVs per section of the same mouse). **D)** average number of *S*.Tm associated with the epithelium. Upper ends of the bars indicate the median. Dotted lines indicate the detection limit. **Panels C-D**) Pooled from 2 or 3 independent experiments for each group: day 1 p.i. (n = 6 mice) and day 3 p.i. (n = 9 mice). Two-tailed Mann Whitney-U tests were used to compare two groups in each panel. P≥0.05 not significant (ns), p<0.05 (*), p<0.01 (**), p<0.001 (***).

It was shown that following an acute *Toxoplasma gondii* gastrointestinal infection of mice, neutrophils can form intraluminal casts that contain commensal outgrowth and prevent spread of pathobionts to the systemic organs [28]. Besides, neutrophils have been shown to interact with luminal *S*.Tm closely during the early stages (at 20h p.i.) of our streptomycin mouse model [1]. Therefore, we speculated that neutrophils might not only be killing luminal pathogens, but also may have a role to reduce *S*.Tm access to the epithelial surface at some phase of the infection, thereby restricting further pathogen invasion events. Fluorescence microscopy of cecal tissue at 18h p.i., day 1 p.i., and 3 p.i. revealed that intraluminal neutrophils and luminal *S*.Tm were in close contact in streptomycin pretreated mice (**Fig 2A**). At day 1 p.i., neutrophils were present in the gut lumen in aggregates of various sizes (ca. 2 cells to 50 cells). We hypothesized that aggregates might prevent pathogen access to the epithelium. To obtain correlative evidence, we analyzed image data as presented in **Fig 2A**, calculated the median number of neutrophils per 63-X field of view and quantified the number of *S*.Tm cells interacting with the epithelium in two scenarios, that is in fields of view with >15 neutrophils vs fields of view with ≤15 neutrophils. Strikingly, the number of *S*.Tm cells in close association with the epithelium was significantly higher in the scenario where there were only a few (≤15) neutrophils in the area in comparison to the scenario with >15 neutrophils (**Fig 2B**). Of further note, we observed that the number of neutrophils in the gut lumen was significantly higher at day 3 p.i. compared to day 1 p.i. (**Fig 2C**). At day 3 p.i., neutrophils assembled into dense structures in front of the epithelium reminiscent of the intraluminal casts reported in other contexts [28]. In accordance with this, the average numbers of epithelium-associated pathogen cells were significantly lower at day 3 p.i. compared to day 1 p.i. (**Fig 2D**).

Neutrophil aggregate formation appeared to start between 18h and 24h p.i.. At both time points, luminal pathogen loads and average numbers of luminal neutrophils were roughly equivalent. However, the number of epithelium associated pathogen cells was significantly higher at 18h p.i. and the neutrophils were present in the gut lumen in individual cells rather than forming aggregates in front of the epithelium, as observed at 24h p.i. or later (**Fig 2C and 2D**). Altogether, our findings suggest that in response to *S*.Tm infection neutrophils are recruited to the gut lumen where they generate large cell aggregates in front of the epithelium and reduce the interaction between *Salmonella* cells and the gut epithelium, starting from day 1 p.i. onwards in the streptomycin pretreated mouse model.

### Neutrophils release extracellular traps (NETs) into the gut lumen in response to acute *Salmonella* infection

**Fig 3:**
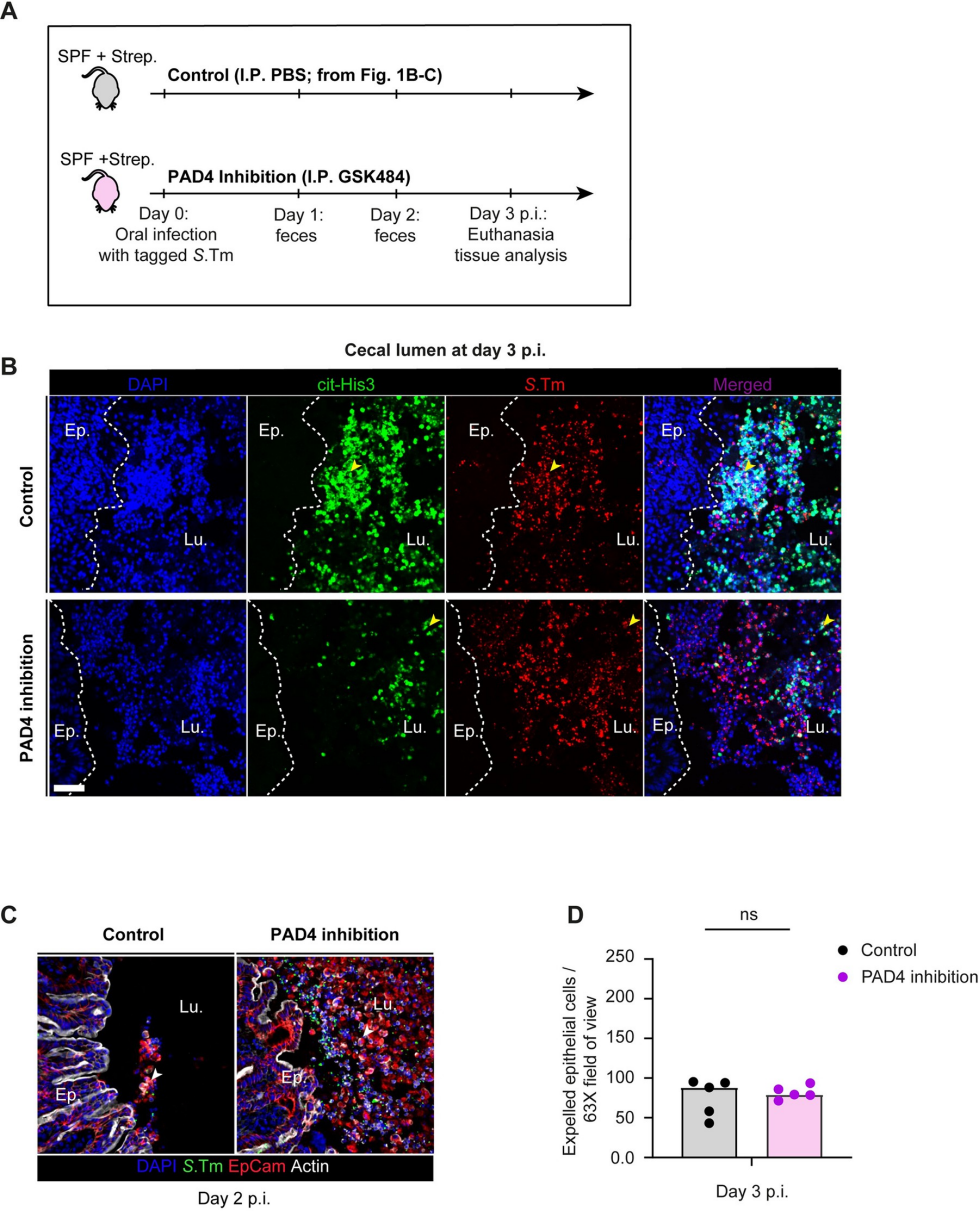
Microscopy analysis of intraluminal NETs at day 3 p.i. **A)** Experimental scheme for **Panels B-D.** Streptomycin pretreated C57BL/6 mice were infected orally with 5x10^7^ CFU of wt *S*.Tm for 3 days. One group (control from **Fig 1B and 1C**) treated with the vector (PBS; black symbols) and the second group with PAD4 inhibitor (GSK484; purple symbols) intraperitoneally (I.P.). **B)** Representative micrographs of cecal tissue sections from mice infected with *S*.Tm, taken at 3 days p.i., stained for NET marker cit-His3 and *Salmonella* LPS. Lu. = Lumen. Ep. = Epithelium. Yellow arrows indicate *S*.Tm associated with the NET marker cit-His3. White dotted lines indicate the epithelial barrier. Scale bar = 50 μm. **C)** Representative micrographs of cecal tissue sections, stained for epithelial marker EpCam and *Salmonella* LPS. Lu. = Lumen. White arrows point at expelled epithelial cells. Scale bar = 50 μm. **D**) Microscopy-based quantification of IECs per 63x field of view (i.e., cells/high power field; hpf). Each data point is the average of 5 fields of view (FOV) per section. Two-tailed Mann Whitney-U tests were used to compare two groups in each panel. p≥0.05 not significant (ns).

Next, we investigated if neutrophil extracellular traps (NETs; [7,8,29]) might help to prevent *S*.Tm access to the gut epithelium. NETs are networks of extracellular host-DNA decorated with antimicrobial peptides. They are released in response to several stimuli and can kill or immobilize the pathogens in the extracellular space [30]. We speculated that the release of NETs into the gut lumen could provide one mechanism by which neutrophils limit further *S*.Tm invasion into the cecum epithelium. Since their discovery two decades ago [29] as a powerful and unorthodox immune defense mechanism, NETs have been implicated not only in the defense against many extracellular pathogens [30], but also in tissue damage and autoinflammatory diseases [5,31,32]. We reasoned that NETs-entrapped *S*.Tm cells might not only be immobilized, but might also be efficiently removed with the fecal flow. NETs are generated during a programmed cell death known as ‘NETosis’, which is initiated by peptidyl arginine deiminase-4 (PAD4, aka Padi4), promoting the formation of citrullinated histone 3 (cit-His3). A previous study showed that PAD4 is necessary for an efficient neutrophil response to the enteric pathogen *Citrobacter rodentium* [33]. Therefore, cit-His3 can be used as a marker for NETs. Our microscopy analysis of intraluminal neutrophil aggregates at day 3 p.i. indeed revealed that parts of these aggregates contained DNA with citrullinated histones (**Fig 3A** and see results below in **Fig 3B**; control). Interestingly, these cit-His3+ regions showed an overlap with regions where *S*.Tm cells were densely localized in the lumen. These results suggest that intraluminal neutrophils can undergo NETosis during acute *Salmonella* infection, and that pathogen cells appear to become entrapped.

Next, we set out to use a NETosis inhibitor to probe the relevance NETosis in our infection model. For this purpose, we used the PAD4 inhibitor, GSK484, shown to effectively reduce the formation of NETs by mouse and human neutrophils [34,35]. Here we asked if PAD4 inhibition could prevent the observed bottleneck of the luminal *S*.Tm population or affect the excessive epithelial cell loss similar to what we observed before (**Fig 1B–1E**). We infected mice as in the experiment described in **Fig 1** using a mixture of untagged and tagged *S*.Tm (1:1000), and treated this experimental group with the PAD4 inhibitor GSK484 (**Fig 3A**; see details in **Materials & Methods**). As untreated controls, we used the control mice from **Fig 1**. Inhibition of PAD4 did, however, only result in a weak non- significant trend in the size of the gut lumen bottleneck while it did not change the pathogen densities in the feces and systemic organs (**S2A–S2D Fig**; evenness score: ≈0.67 vs 0.46, p = 0.44). Microscopy images of the infected cecum revealed that neutrophil aggregates in the gut lumen still contained detectable cit-His3+ DNA also in mice treated with the inhibitor (**Fig 3B**). It should be noted that strategies to pharmacologically prevent NETosis are still under investigation [36], and it seems clear that our approach only partially prevented NETosis in the gut lumen of our *in vivo*-infection model. Therefore, these results should be considered as preliminary for a role of NETs in defense against *S*.Tm. Nevertheless, the partial inhibition of NETosis resulted in elevated epithelial cell loss at day 2 p.i. (**Fig 3C**). However, by day 3 p.i., we could no longer detect any PAD4 effect on the number of expelled epithelial cells (**Fig 3D**). Taken together, our preliminary results show that intraluminal neutrophils are closely interacting with *Salmonella* during the acute infection, and that these neutrophils are positive for the NETosis marker cit-His3. This suggests that the release of NETs into the gut lumen occurs. However, future work will be needed to establish if this contributes to epithelial defense during *S*.Tm infection.

### Wild type *S*.Tm loads in the gut tissue contribute to the epithelial damage at day 3 p.i. in streptomycin pretreated mice

So far, our approaches suggested a correlation between neutrophil depletion and exacerbated epithelium response to the insult. However, they remained insufficient to establish that the protection of the epithelial barrier is attributable to intraluminal neutrophils. This can partly be explained by the limitations of our experimental infection model. Wt *S*.Tm utilizes TTSS-1 and -2 (encoded on *Salmonella* Pathogenicity Islands (SPI)-1 and -2, respectively) to trigger gut disease [2]. In our mouse model, SPI-2 enables not only long-term survival of the pathogen at systemic sites (which becomes very prominent after days 3–4 p.i.), but is also promotes *S*.Tm colonization of the lamina propria. Importantly, the gut-luminal and the gut-tissue-lodged *S*.Tm populations are both interacting with neutrophils. The data presented above could not discern if the enhanced epithelial damage in neutrophil-depleted mice is attributable to exacerbated attack by the gut-luminal or over-shooting immune responses elicited by the tissue-lodged *S*.Tm population.

**Fig 4:**
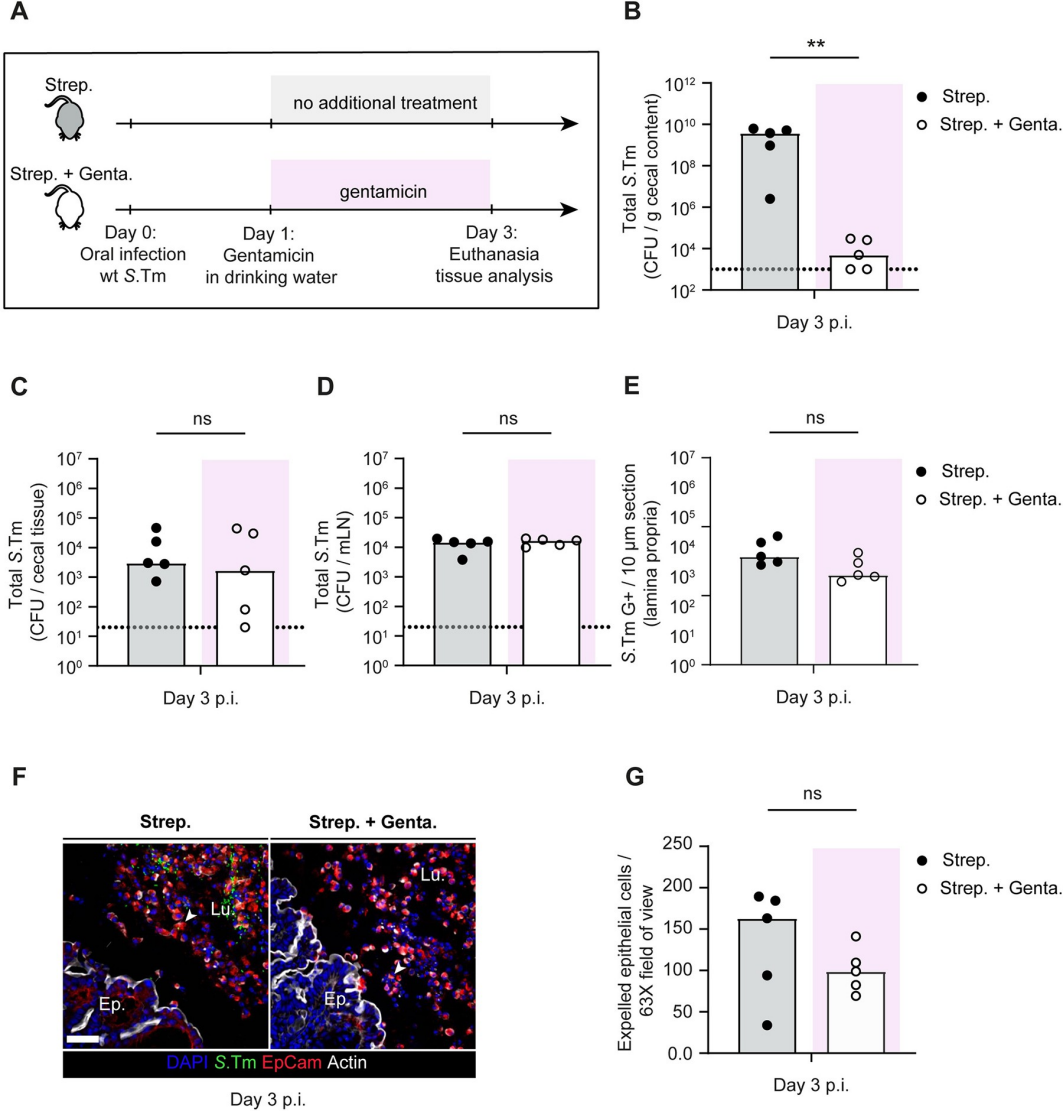
Investigation of the contribution of wt pathogen loads in the gut tissue to epithelial cell loss. **A)** Experimental scheme. Streptomycin pretreated C57BL/6 mice were infected orally with 5x10^7^ CFU of wt *S*.Tm for 3 days. Mice were divided into two groups: 1) without gentamicin, 2) with gentamicin in drinking water starting from day 1 p.i.. Total *S*.Tm pathogen loads **B)** in cecal content, **C)** in the cecal tissue, and **D)** mLN at day 3 p.i. in each group. **E)** Microscopy-based quantification of *S*.Tm cells residing in the lamina propria. **F)** Representative micrographs of cecal tissue sections, stained for epithelial marker EpCam and *Salmonella* LPS. Lu. = Lumen. Ep. = Epithelium. White arrows point at expelled epithelial cells. Scale bar = 50 μm. **G**) Microscopy-based quantification of luminal IECs per 63x field of view (i.e., cells/high power field; hpf). Each data point is the average of 5 fields of view (FOV) per section. Upper ends of the bars indicate the median. **Panels B-G**) Pooled from total 2 independent experiments; group-1 (n = 5 mice), group-2 (n = 5 mice). Two-tailed Mann Whitney-U tests were used to compare two indicated groups in each panel. p≥0.05 not significant (ns), p<0.01 (**).

First, we tested if wild type *S*.Tm growth in the lamina propria is sufficient to drive epithelium damage. This seemed plausible, as our results from **Fig 3C and 3D** (control) suggest that epithelium damage is clearly observable even in control mice at day 3 p.i., highlighting that wt *S*.Tm virulence leads to enteropathy in this model even in the presence of neutrophils. To test if the pathogen cells in the gut tissue are sufficient to drive epithelial cell loss at day 3 p.i., we infected antibiotic pretreated mice with wt *S*.Tm. In the experimental group, we cleared luminal pathogen cells from day 1 p.i. onwards by supplementing the drinking water with gentamicin, an antibiotic which acts locally in the gut lumen but does not cross the epithelium (experimental scheme; **Fig 4A**). Gentamicin treatment resulted in a significant decrease (i.e.,100,000-fold) of luminal pathogen densities at day 3 p.i. (**Fig 4B**; from 10^9^ to ca. 10^4^ CFU / g feces). In contrast, pathogen densities in the cecal tissue, mLN, and the lamina propria did not differ between the two groups (**Fig 4C–4E**). This confirmed that gentamicin selectively kills luminal *S*.Tm cells and that wt *S*.Tm loads in the underlying gut tissue and systemic sites remain high without further invasion events from the luminal side. Next, we investigated the degree of epithelial damage in both groups of mice. Strikingly, we observed severe epithelial cell loss in both groups, with only modestly lower numbers of expelled epithelial cells in the gentamicin treated group (**Fig 4F and 4G**). These observations suggest that wt pathogen cells located in the lamina propria (or at systemic sites) contribute to the epithelial cell loss at day 3 p.i. and that this cell loss happens also when luminal pathogen densities are suppressed.

### Intraluminal neutrophils limit continuous insults to the epithelium and prevent massive epithelial cell loss

To specifically study the consequences of lack of gut-luminal neutrophils on epithelial responses to *S*.Tm at mature stages of infection, we infected antibiotic pretreated mice with a SPI-2 mutant (*S*.Tm^*ssaV*^). This mutant strain is still able to trigger SPI-1 -dependent invasion and gut inflammation and the enteropathy is about as pronounced as for wt *S*.Tm infections, at least during the first 2 days of infection [2]. However, in contrast to wt *S*.Tm, the *S*.Tm^*ssaV*^ infection does not progress into a typhoid like disease at later stages and colonizes the cecum lamina propria with reduced efficiency [2]. With this model, we were able to minimize the contribution of pathogen cells residing in deeper tissues to epithelial cell expulsion or disruptive shedding, and study epithelial attack from the lumen in isolation. To this end, we pretreated C57BL/6 mice with ampicillin [37], instead of streptomycin, and infected with 5x10^7^ CFU of *S*.Tm^*ssaV*^ for 3 days. Of note, *S*.Tm^*ssaV*^ gut colonization does neither differ between the two treatments (streptomycin or ampicillin) nor from wt *S*.Tm gut colonization (**S3A-S3C Fig**). However, ampicillin pretreatment leads to more robust inflammation kinetics which enhances our ability to study the role of intraluminal neutrophils when using the SPI-2 mutant pathogen (**S3D Fig**).

**Fig 5:**
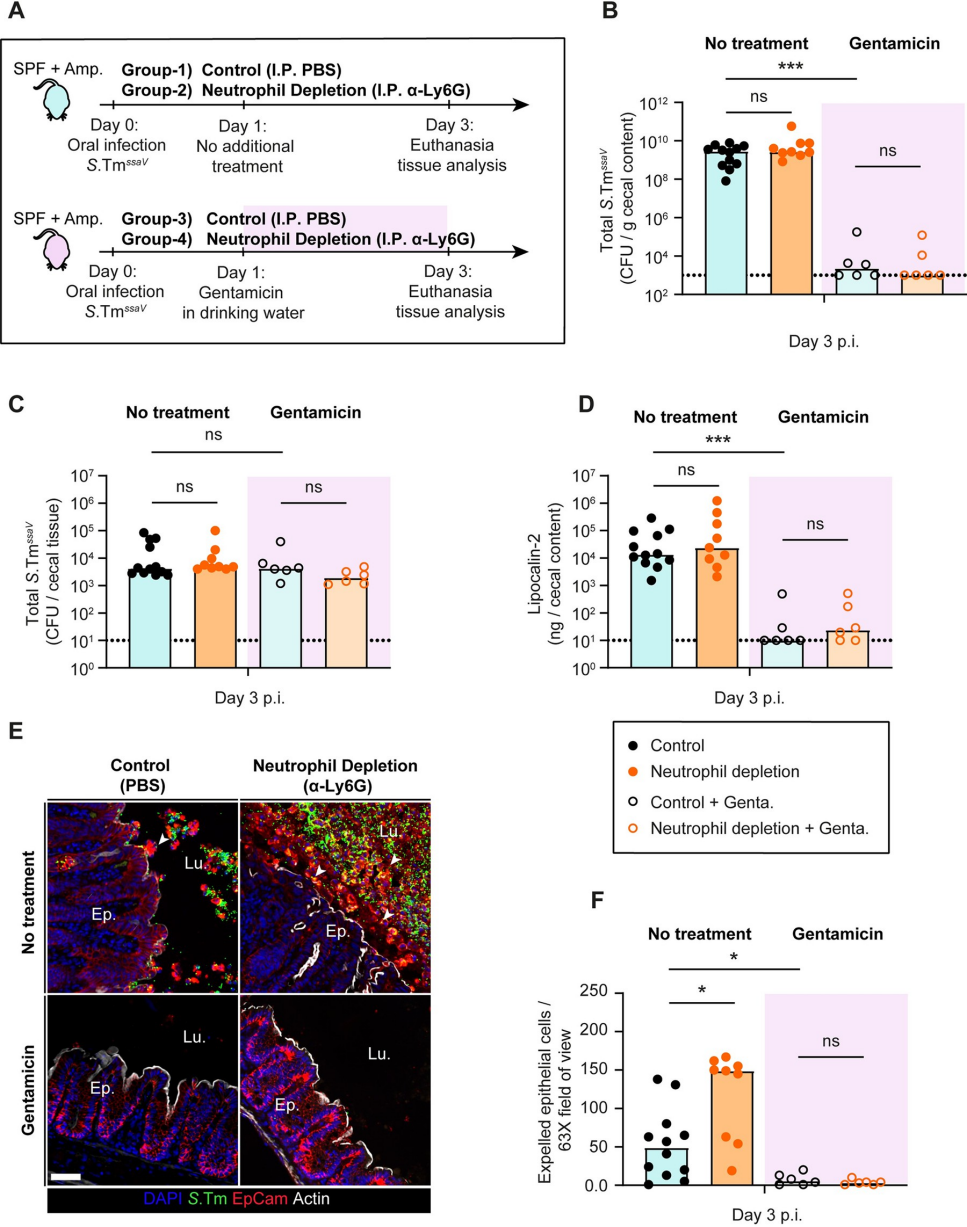
Investigation of the role of intraluminal neutrophils in a mouse model with reduced systemic disease. **A)** Experimental scheme for **Panels B-F**. Ampicillin pretreated C57BL/6 mice were infected orally with 5x10^7^ CFU of *S*.Tm^*ssaV*^ for 3 days. Mice were divided into four groups: 1) Control (I.P. PBS; black filled symbols) without gentamicin, 2) Neutrophil depletion (I.P. α-Ly6G; orange filled symbols) without gentamicin, 3) Control (I.P. PBS; black empty symbols) with gentamicin in drinking water starting from day 1 p.i., and 4) Neutrophil depletion (I.P. α-Ly6G; orange empty symbols) with gentamicin in drinking water starting from day 1 p.i. Total *S*.Tm^*ssaV*^ pathogen loads **B)** in cecal content, **C)** in the cecal tissue at day 3 p.i. in each group. **D)** Quantification of gut inflammation by Lipocalin-2 levels in cecal content. **E)** Representative micrographs of cecal tissue sections, stained for epithelial marker EpCam and *Salmonella* LPS. Lu. = Lumen. Ep. = Epithelium. White arrows point at expelled epithelial cells. Scale bar = 50 μm. **F**) Microscopy-based quantification of luminal IECs per 63x field of view (i.e., cells/high power field; hpf). Each data point is the average of 5 fields of view (FOV) per section. Upper ends of the bars indicate the median. **Panels B-F**) Pooled from total 4 independent experiments; at least 2 for each group: Group-1 (n = 12 mice), group-2 (n = 9 mice), group-3 (n = 6 mice), group-4 (n = 6 mice). Two-tailed Mann Whitney-U tests were used to compare two indicated groups in each panel. p≥0.05 not significant (ns), p<0.05 (*), p<0.001 (***).

To probe the significance of intraluminal neutrophils in ampicillin pretreated mice infected with S.Tm^*ssaV*^, we compared the effects of neutrophil depletion to a scenario where we removed the luminal pathogen population after day 1 p.i. by supplementing the drinking water with gentamicin as described above (experimental scheme; **Fig 5A**). We reasoned that if the exacerbated epithelial response (observed in **Fig 1D–1E**) is caused by continuous high-level pathogen invasion from the luminal side, the gentamicin treatment should alleviate this outcome. At day 3 p.i., *S*.Tm^*ssaV*^ was undetectable in the cecal content in all groups undergoing gentamicin treatment while the pathogen densities stayed high in feces and cecal content throughout the experiment in groups without gentamicin (**Figs 5B and S3E**). On the other hand, removal of the luminal pathogen population by gentamicin did not affect the pathogen loads in the cecal tissue and the systemic organs at day 3 p.i. in either of the groups infected with *S*.Tm^*ssaV*^ (**Figs 5C and S3F**, open symbols). This provided an ideal set-up to test the role of intraluminal neutrophils. Second, we assessed if the absence of the luminal pathogen population influenced the enteric disease kinetics. Strikingly, concentrations of Lipocalin-2 (a general marker for gut inflammation) were significantly reduced at day 3 p.i. in the groups treated with gentamicin in the drinking water (both control and neutrophil depletion with gentamicin treatment; **Fig 5D**). This suggested that the pathogen population in the gut lumen significantly contributes to the maintenance of gut inflammation. Finally, we explored the effect of neutrophil depletion on epithelial pathology and integrity. To this end, we compared the number of expelled epithelial cells in control vs neutrophil depleted mice in the absence or presence of gentamicin treatment (**Fig 5E and 5F**). In the group without gentamicin treatment, neutrophil depletion caused a significant elevation of expelled epithelial cells compared to the mock-depleted control, similar to as we observed in **Fig 1D** (**Fig 5E and 5F**). Furthermore, mRNA expression of *Ccl2*, *Il6*, and *Mmp8* were elevated in neutrophil depleted animals, suggesting an overstimulation of the cecal epithelium (**S3G Fig**). Similarly, the epithelial cells again appeared to dislodge from the mucosa in an uncontrolled manner and in big aggregates in neutrophil-depleted animals (**Fig 5E**). In stark contrast, neutrophil depletion did not lead to an elevated frequency of epithelial cell expulsion in the group receiving gentamicin treatment (**Fig 5E and 5F**). In fact, the gentamicin treatment reduced the numbers of expelled epithelial cells in the lumen down to baseline at 3 days p.i. (compare open symbols to black filled symbols in **Fig 5F**). This effect was observed in mice with and without neutrophil depletion. Upon gentamicin treatment, the epithelium ultrastructure also recovered, as judged by clearly defined and organized crypt structures. These findings reveal that epithelial invasion events from the lumen at mature stages of *S*.Tm^*ssaV*^ infection continue to fuel epithelial cell expulsion, and substantiate that intraluminal neutrophils provide a crucial barrier to reduce the frequency of these events so that epithelial barrier integrity can be retained.

### The lack of intraluminal neutrophil defense is detrimental for the epithelium in mice lacking a resident gut microbiota

Previous studies highlighted microbiota mediated colonization resistance, mucus secretion by goblet cells, antimicrobial peptides secretion, epithelial NAIP/NLRC4 inflammasome, and neutrophil infiltration into the gut lumen as potentially complementary factors protecting the epithelium from pathogen attack. How these various protective systems are integrated with each other remains poorly understood, but it appears conceivable that defects in any of them will result in a heavier reliance on the others, e.g. a more prominent dependence on the luminal neutrophil defense predicted in animals lacking other protection factor(s). One good example of such a scenario would be the absence of resident microbiota, which has been shown to exacerbate *S*.Tm-induced colitis in mice [19].

**Fig 6:**
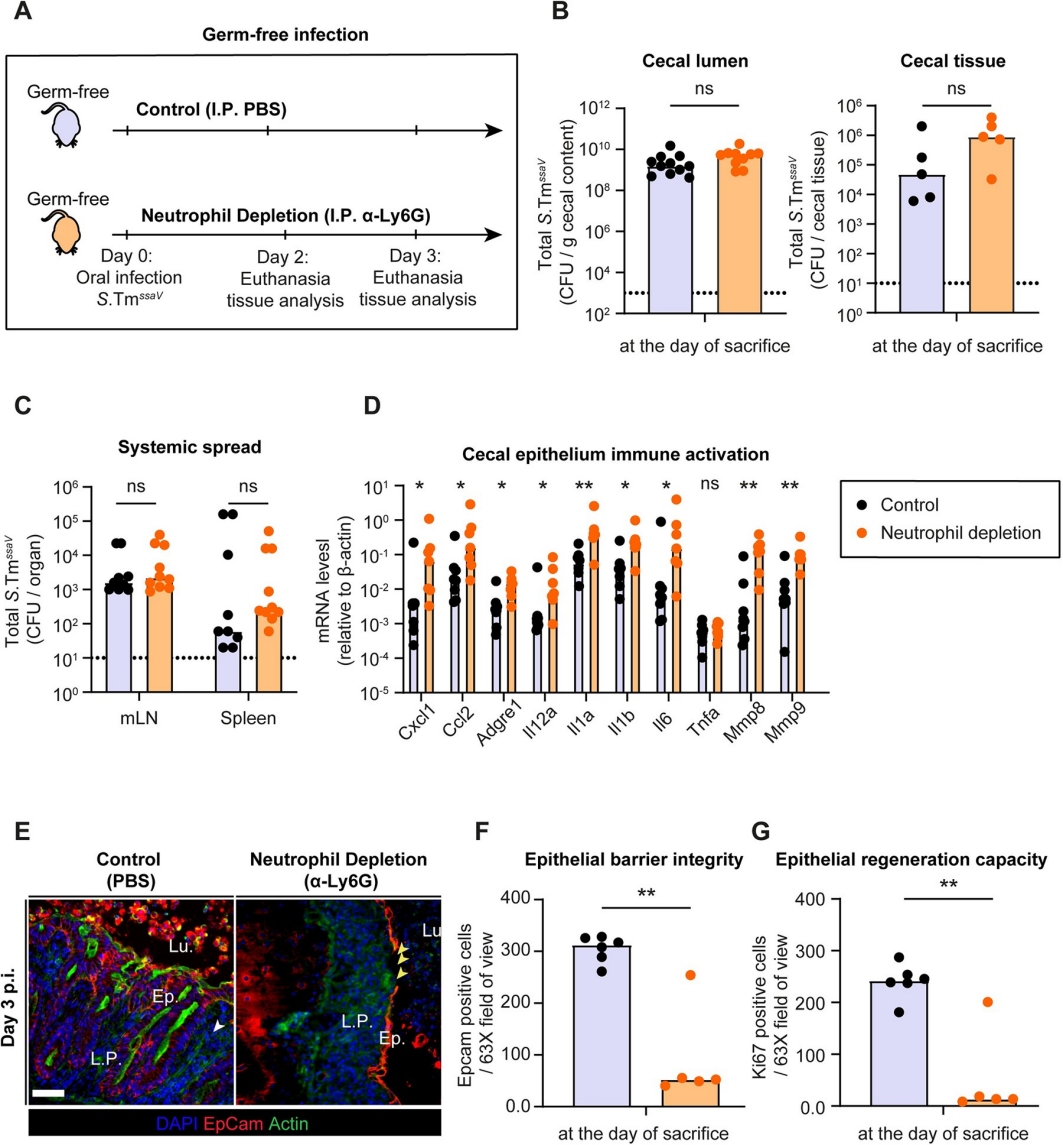
Consequences of neutrophil depletion on epithelial health during *S*.Tm^*ssaV*^ infection of germ-free mice. **A)** Experimental scheme for **Panels B-G**. C57BL/6 germ-free mice were infected orally with 5x10^7^ CFU of *S*.Tm^*ssaV*^ for 2 or 3 days (mice were euthanized at either day according to the health status of the neutrophil-depleted mice). Mice were divided into 2 groups: 1) Control (I.P. PBS; black filled symbols), 2) Neutrophil depletion (I.P. α-Ly6G; orange filled symbols). Total *S*.Tm^*ssaV*^ pathogen loads **B)** in cecal content and cecal tissue, **C)** in mLN and spleen (pooled data from day 2 and 3 p.i.) in each group. **D)** Quantification of mRNA expression levels in the cecal tissue by qRT-PCR, for genes involved in immune response to acute *Salmonella* infection and genes involved in tissue remodelling. Results are represented relative to β-actin mRNA levels. **E)** Representative micrographs of cecal tissue sections, stained for epithelial marker EpCam and *Salmonella* LPS. Lu. = Lumen. Ep. = Epithelium. L.P. = Lamina Propria. Yellow arrows point at regions with gaps in the epithelial barrier. Scale bar = 50 μm. Microscopy-based quantification of **F**) IECs remaining in the tissue per 63x field of view and **G)** Ki67+ cells per 63x field of view (i.e., cells/high power field; hpf). Each data point is the average of 5 fields of view (FOV) per section. Upper ends of the bars indicate the median. Data pooled from total 5 independent experiments. Data reported in **Panel B-G** is pooled from mice euthanized at day 2,3 and 4 p.i. as results were indistinguishable after day 2 p.i.. **Panel C**: control (n = 5 from day 2 and n = 4 from day 3), neutrophil depletion (n = 5 from day 2 and n = 5 from day 3). **Panel D**: control (n = 2 from day 2, n = 4 from day 3, and n = 2 from day 4), neutrophil depletion (n = 2 from day 2 and n = 5 from day 3). **Panel F-G**: control (n = 2 from day 2, n = 2 from day 3, and n = 2 from day 4), neutrophil depletion (n = 2 from day 2 and n = 3 from day 3). Two-tailed Mann Whitney-U tests were used to compare two indicated groups in each panel. p≥0.05 not significant (ns), p<0.05 (*), p<0.01 (**).

To test this hypothesis, we infected germ-free C57BL/6 mice with 5x10^7^ CFU of *S*.Tm^*ssaV*^ and compared neutrophil-depleted mice to control mice (**Fig 6A**). Since germ-free mice lack resident microbiota, they are more susceptible to *Salmonella* infection [38]. Importantly, the epithelial regeneration which is critical for maintaining epithelial integrity during an acute *S*.Tm infection is also delayed in germ free mice [19]. These features allowed us to scrutinize the role of intraluminal neutrophils even more readily than in antibiotic pretreated mice associated with a conventional microbiota. In germ-free mice, *S*.Tm^*ssaV*^ colonized the gut to the carrying capacity in both groups and *S*.Tm^*ssaV*^ was shed into the feces at very high numbers until day 3 p.i. (≈10^9^ CFU / g feces; **Fig 6B**). Moreover, neutrophil depletion did not significantly affect the cecal tissue loads or systemic spread of the pathogen (**Fig 6B and 6C**). However, mRNA expression analysis of cecal tissue revealed a striking difference between depleted and non-depleted mice. Many genes encoding pro-inflammatory cytokines and chemokines associated with the acute *Salmonella* infection were induced to higher levels in the cecum of neutrophil-depleted mice compared to the controls (**Fig 6D**). This supported our hypothesis that neutrophil depletion leads to an overstimulation of the mucosal immune defense.

Next, we asked whether this hyper-activation of the mucosal immune responses is detrimental to the epithelial barrier. To test this, we first analysed the mRNA expression levels of matrix-metalloproteinases (Mmp-8 and -9) associated with inflammation-linked tissue remodelling [39]. Indeed, the neutrophil-depleted group showed an elevated mRNA expression of these genes (**Fig 6D**). Second, we analysed the epithelial barrier integrity and the epithelial regeneration responses in these mice. Strikingly, fluorescence microscopy of cecal tissue at day 2 and 3 p.i. revealed that neutrophil depletion caused a severe loss of epithelial cells and formation of gaps in the barrier (**Fig 6E and 6F**). The number of epithelial cells per crypt was also dramatically reduced and organized crypt structures were entirely lost in some mice (**Fig 6E and 6F**). To test if this loss of epithelial barrier was associated with a reduced capacity of the epithelial cells to divide, we analysed the fraction of cells expressing the proliferation marker Ki67. Indeed, actively dividing cells were nearly absent in neutrophil-depleted groups while the active division and crypt hyperplasia was apparent in the control mice infected with *S*.Tm^*ssaV*^ (**Figs 6G and S4A**). In control mice, more than 90% of the epithelial cells of a crypt were positive for Ki67. Therefore, the lack of gut microbiota appears to lead to an overwhelming attack on the epithelium during *S*.Tm^*ssaV*^ infection, which is counteracted by neutrophils.

Finally, we hypothesized that colonization resistance conferred by the microbiota might obliviate the need for neutrophil defenses in preventing the epithelium disruption in the infected gut. This is of course trivial in the case of C57BL/6 mice harboring a complex microbiota that prevents gut-luminal pathogen growth and enteropathy right from the start [11]. We could however perform a pilot experiment in C57BL/6 mice associated with the OligoMM^12^ microbiota, which confer partial colonization resistance and show overt enteropathy only by days 3–4 after oral *S*.Tm infection [40]. Strikingly, neutrophil depletion did not exacerbate the epithelial damage in OligoMM^12^ mice, further highlighting the complementary epithelium-protective roles of the resident microbiota and neutrophil defence (**S5A–S5F Fig**).

Taken together, our results suggest that intraluminal neutrophils and microbiota may cooperate in protecting epithelial integrity during an *S*.Tm infection. In absence of microbiota, the neutrophils have a particularly pronounced protective function. In the presence of intraluminal neutrophils, epithelial integrity can be maintained by rapid epithelial proliferation even in germ-free mice, while the depletion of neutrophils is detrimental to the epithelial barrier integrity.

## Discussion

Since neutrophils are central players exerting diverse effector functions during the initial immune response to infections, a full understanding of neutrophil functions in the course of diverse infectious diseases is crucial. In the case of *Salmonella* gut infection, the role of neutrophils has not been fully elucidated. Here we tackled this question and carefully analysed the consequences of neutrophil depletion (with α-Ly6G) during the infection with *S*.Tm in multiple mouse models for *Salmonella* gut infection. Our findings provide evidence that intraluminal neutrophils provide a barrier in front of the epithelium during a critical stage of the infection and that neutrophil function is essential for preventing excessive epithelial cell loss, maintaining epithelial regeneration at a rate that preserves barrier integrity, and thus avoiding epithelium disruption (**S6A and S6B Fig**).

In this report, we present an epithelium-protective role of neutrophils in different variants of the mouse infection model for acute Salmonellosis (**Figs 1, 5 and 6**). The streptomycin pretreated mouse model is often used to study acute *Salmonella* infection. It employs wt *S*.Tm (i.e., intact SPI-1 and SPI-2) that elicits enteropathy and is further characterized by the successive development of a typhoid-like disease, which can overwhelm Nramp-1-negative mouse strains at later time points (beyond days 5–6 p.i.). This parallel systemic infection makes it harder to study the role of immune effector mechanisms that are mounted after the early NAIP/NLRC4-dependent epithelial cell expulsion response [22]. Here, by combining ampicillin pretreatment and germ-free models with a SPI-2 mutant (which fails to grow to high levels at systemic sites), we could resolve the defenses protecting against luminal pathogen insults after day 1 p.i.. Because the mutant pathogen is defective in its ability to grow at systemic sites, we could focus on the luminal events, providing us with vital information regarding the neutrophils`function in the gut lumen. Furthermore, this model revealed that the immune response to the pathogen is highly regulated as indicated by the rapid reduction of Lipocalin-2 secretion upon antibiotic treatment; **Fig 5D**). We believe that, although we use a mutant strain in a murine model, these findings may generalize to *Salmonella* gut infections in the wild, which rarely result in detectable systemic infection [41].

Our findings highlight the multi-layered nature of the mucosal defence against enteric *S*.Tm infection. In our work, the degree of epithelial damage was different depending on the type of infection model applied. These differences were particularly striking when comparing the ampicillin pretreated mice in **Fig 5** with germ-free mice studied in **Fig 6**. During infection of ampicillin pretreated conventional mice with *S*.Tm^*ssaV*^, neutrophil depletion caused excessive epithelial cell loss, but the crypts stayed intact without any larger gaps in the epithelium. In contrast, neutrophil-depleted germ-free mice developed pronounced epithelial damage and essentially a full collapse of crypt ultrastructure. Conversely, the neutrophil depletion did not have any discernible effect on epithelial integrity in the pilot experiment in C57BL/6 mice associated with the OligoMM^12^ microbiota (**S5 Fig**). This highlights the importance of resident microbiota in complementing the here described intraluminal neutrophil defense. Presumably, this is attributable to sub-acute stimulation of innate immune responses by molecular patterns derived from the resident microbiota [42]. Such sub-acute stimulation might enhance the regenerative capacity of the gut epithelium and thereby prevent pronounced epithelium disruption even if neutrophils are depleted. The delayed gut colonization- and disease kinetics in OligoMM^12^ mice might also be involved. Therefore, the effects of antibiotic-mediated depletion of the gut microbiota should be studied in more detail to gain a deeper understanding of the role of continued sub-acute stimulation of mucosal defences by the microbiota, their effects on the infection kinetics or their loss during antibiotic treatment. Several alternative mouse models which permit *S*.Tm gut infections in the face of a pre-existing microbiota, such as low-complexity microbiota-bearing mice (similar to OligoMM^12^) or transient diet shift models, have been proposed recently [40,43]. We hypothesize that studying the host immune responses to *Salmonella* infection in models with milder microbiota perturbation can provide us with important new insights regarding microbiota–innate immune defense integration.

Several reports highlighted that NETs (that is host DNA decorated with antimicrobial agents) not only constitute a potent antimicrobial mechanism, but that NETs also can cause tissue damage and elicit autoimmunity [30]. Our observations hint that the intestinal lumen may be a unique site for deploying NETs as a specific anti-infective defense, without risking NET-mediated tissue damage or autoimmunity. Our results show that aggregates of neutrophils in the gut lumen are positive for one of the NETosis markers, citrullinated histone 3. Further work with alternative NET inhibition approaches may shed light on the function of NETs in epithelial defense. Nevertheless, our study does substantiate that neutrophils form large intraluminal aggregates that physically and/or biochemically shield the epithelium from sustained pathogen attack, similar to earlier work showing that neutrophils can confine pathobiont expansion by forming intraluminal casts [28].

In summary, we have presented evidence for the protective role of intraluminal neutrophils during key stages of acute *Salmonella* infection in mice, by providing a protective barrier against luminal pathogen attack and hence giving the epithelium time to recover through crypt cell proliferation. Of note, our findings are limited to the murine models described in this study, and further research will be needed to assess if intraluminal neutrophils function in a similar fashion in other relevant scenarios and hosts. Our findings provide a basis for future research to disentangle the molecular mechanism(s) by which neutrophils exert this protective role, how this integrated with microbiota-dependent protection and to explore if these observations can be broadly applied across the diversity of enteroinvasive infections.

## Materials and methods

### Ethics statement

All animal experiments were approved by the local governing body, Kantonales Veterinäramt Zürich (under the licence numbers: ZH193/2016, ZH158/2019 and ZH108/2022).

### Bacterial strains used in this study

In all experiments, *Salmonella* Typhimurium SL1344 (*S*.Tm;SB300; SmR) or the indicated *ssaV* mutant version (*S*.Tm^*ssaV*^; M2730; AmpR) were used. [44,45]. WITS-tags were introduced into *S*.Tm by P22 phage transduction and subsequent selection on kanamycin. The presence of the correct WITS-tag was confirmed by PCR using tag-specific primers [14,46]. For *in vivo* mouse infections, bacteria were grown in lysogeny broth (LB) containing the appropriate antibiotics (50 μg/ml streptomycin (AppliChem); 15 μg/ml chloramphenicol (AppliChem); 50 μg/ml kanamycin (AppliChem); or 100 μg/ml ampicillin (AppliChem)) at 37°C for 12h and sub-cultured in 1:20 LB without antibiotics for 4h. Cells were washed and re-suspended in cold PBS (BioConcept).

### Mouse infections

C57BL/6 mice with different microbiota complexity (germ-free and specific pathogen free (SPF)) were kept in individually ventilated cages of the ETH Zürich mouse facility (EPIC and RCHCI). Infection experiments in antibiotic pretreated mice were done according to the previously-described Streptomycin mouse model for *S*.Tm oral infection [11]. Briefly, the mice were pretreated with 25mg of streptomycin by oral gavage 24h prior to infection and infected on day 0 by oral gavage with an inoculum of 5x10^7^ CFU *S*.Tm. Infections in **Fig 4** followed the same protocol but employed a pretreatment with 20mg of ampicillin and *S*.Tm^*ssaV*^ oral infection. Germ-free mice infections were done similarly but without any pretreatment. Experiments were performed with 8–12-week-old male or female mice. The sample-size was not pre-determined, and mice were randomly assigned to groups.

Mice were monitored daily, and organs were harvest at the indicted time points. Feces were collected at the indicated time points and where necessary, cecal tissue and mLN were harvested at the end of the infection. For cecal tissue plating, the gentamicin protection assay was used in which the tissue is treated with gentamicin to clear extracellular bacteria. Cecal tissue was cut longitudinally, washed rapidly in PBS (3x), incubated for 45-75min in PBS/400μg/ml gentamicin Sigma-Aldrich) at RT, and washed extensively (3x 30s) in PBS before plating. For plating, the samples were homogenized with a steel ball in a tissue lyser (Qiagen) for 2 minutes at 25Hz frequency (cecal tissue 3 minutes at 30Hz). The homogenized samples were diluted in PBS, plated on MacConkey (Oxoid) plates supplemented with the relevant antibiotic(s), and placed at 37°C overnight. Colonies were counted the next day and represented as to CFU / g content.

For *in vivo* depletion of neutrophils, anti-Ly6G (BioXCell, 1A8) was injected intraperitoneally at each day starting at pretreatment (In **Fig 1** 500μg/mouse, in **Figs 4 and 5** 250 μg/mouse). For PADK4 inhibition experiments, the inhibitor GSK484 (500μg/mouse; in 10% DMSO; Cayman Chemical) was injected intraperitoneal daily starting at pretreatment.

### qRT-PCR

Cecal tissue sections were snap-frozen in RNAlater solution (Thermo Fisher Scientific) after extensive washing of the content in PBS and stored in -80°C until further analysis. Total RNA was extracted using RNeasy Mini Kit (Qiagen) according to manufacturer’s instructions and converted to cDNAs employing RT^2^ HT First Strand cDNA Kit (Qiagen). qPCR was performed with FastStart Universal SYBR Green Master reagents (Roche) and Ct values were recorded by QuantStudio 7 Flex FStepOne Plus Cycler. Primers were designed using the NCBI primer-designing tool (see **Table 1**) or ordered as validated primers from Qiagen. The mRNA expression levels were plotted as relative gene expression to β-actin (2^-ΔCt)^) and comparisons are specified in the figure captions.

**Table 1:** Primer Sequences used for real time qRT-PCR.

Gene Name		Primer Sequence (5`➔3`)
β-actin (mouse)	**F** **R**	AGAGGGAAATCGTGCGTGAC CAATAGTGATGACCTGGCCGT
Cxcl1 (mouse)	**F** **R**	CCGCTCGCTTCTCTGTGC CTCTGGATGTTCTTGAGGTGAATC
Cxcl2 (mouse)	**F** **R**	CGCCCAGACAGAAGTCATAG TCCTCCTTTCCAGGTCAGTTA
Il12a(mouse)	**F** **R**	TGTGGGAGAAGCAGACCCTTA GGGTGCTGAAGGCGTGAA
Adgre1 (mouse)	**F** **R**	CTTTGGCTATGGGCTTCCAGTC GCAAGGAGGACAGAGTTTATCGTG
Il1b (mouse)	**F** **R**	GCAACTGTTCCTGAACTCAACT ATCTTTTGGGGTCCGTCAACT
Il6 (mouse)	**F** **R**	CCTCTGGTCTTCTGGAGTACC ACTCCTTCTGTGACTCCAGC
Il1a (mouse)	**F** **R**	Qiagen
Tnfa (mouse)	**F** **R**	ATGAGCACAGAAAGCATGA AGTAGACAGAAGAGCGTGGT
Mmp8 (mouse)	**F** **R**	Qiagen
Mmp9 (mouse)	**F** **R**	Qiagen

### Lipocalin-2 ELISA

Lipocalin-2 ELISA (R&D Systems) was performed to determine gut inflammation from fecal samples according to the manufacturer’s instructions. Fecal pellets were suspended in sterile PBS (BioConcept), diluted 1:20, 1:400 or undiluted, and the concentrations were determined by curve fitting using Four-Parametric Logistic Regression.

### Immunofluorescence

Cecal tissue sections from mice were carefully dissected, fixed with 4% paraformaldehyde, saturated in 20% sucrose/PBS, and snap-frozen in Optimal Cutting Temperature compound (OCT, Tissue-Tek). Samples were stored in -80°C freezer until further analysis. Samples to be stained were cut in 10μm cross-sections and mounted on glass slides (Superfrost++, Thermo Scientific). For staining, cryosections on the glass slides were air-dried, rehydrated with PBS and permeabilized using a 0.5% TritonX-100/PBS solution. Sections were blocked using 10% Normal Goat Serum (NGS)/PBS before staining with primary and secondary antibodies. The following antibodies and dilutions were used for the staining of different samples: 1:200 EpCam/CD326 (clone G8.8, Biolegend), 1:200 α-*S*.Tm LPS (O-antigen group B factor 4–5, Difco), 1:200 α-Ki67 (ab15580, Abcam Biochemicals), or 1:200 α-Ly6B.2 clone 7/4, BioRad) in combination with the respective secondary antibodies, i.e α-rabbit-AlexaFluor488 (Abcam Biochemicals), α-rat-Cy3 (Jackson), fluorescent probes, i.e. CruzFluor488-conjugated Phalloidin (Santa Cruz Biotechnology), AlexaFluor647-conjugated Phalloidin (Molecular Probes), and/or DAPI (Sigma Aldrich). Stained sections were then covered with a glass slip using Mowiol (VWR International AG) and kept in dark at room temperature (RT) over night. For confocal microscopy imaging, a Zeiss Axiovert 200m microscope with 10-100x objectives or a spinning disc confocal lased unit (Visitron) with 10-100x objectives were used. Images were processed or analyzed with Visiview (Visitron) and/or ImageJ. Manual quantification was done blindly on two different sections (3 to 5 regions per section) from the same mouse according to the indicated parameters. The number of neutrophils per 63X field of view were counted on epithelium where half of the field included the lumen close to the epithelium to include freshly transmigrated neutrophils. Average crypt sizes were determined by counting the number of EpCAM positive cells per one crypt structure. Epithelial gaps were defined as the mucosal regions were where content of the lumen was in direct contact with the lamina propria. The epithelium associated *S*.Tm cells were counted by scanning the area in 10 μm close proximity to epithelium on the luminal side.

### Statistical analysis

Whenever applicable, the two-tailed Mann Whitney-U test was used to assess statistical significance as indicated in the figure legends. GraphPad Prism 8 for Windows was used for statistical testing. Evenness indices were calculated as previously described [14].

## Supporting information

S1 Fig Consequences of neutrophil depletion with α -Ly6G on luminal pathogen loads, systemic spread, and epithelial cell expulsion (related to Fig 1). **A)** Streptomycin pretreated C57BL/6 mice were infected orally with 5x10^7^ CFU of wild-type *S*.Tm (SL1344) for 3 days. One group (control) was treated with the vector (PBS; black symbols) and the second group with α-Ly6G (orange symbols) intraperitoneally (I.P.). *S*.Tm pathogen loads **A)** in feces until day 3 p.i. (CFU / g) and **B)** in systemic organs at day 2 p.i. (CFU / organ). **C)** Relative ranks of the tagged *S*.Tm strain abundances in feces at day 1 p.i.. **D)** Evenness score at day 1 p.i. **E-F)** *S*.Tm pathogen loads **E)** in the cecal content at day 2 and 3 p.i. (CFU / g) and **F)** in systemic organs (mLN, spleen, and liver) at day 3 p.i. (CFU / organ). Lines or upper ends of the bars indicate the median. Dotted lines indicate the detection limit. **G)** *S*.Tm pathogen loads in cecal tissue (CFU / g). **Panel A**) Pooled from 9 independent experiments for each group: number of mice each day differs as experiments terminated at day 1, 2 or 3 p.i., but n = at least 14 at each day. **Panel B**) Pooled from 4 independent experiments for each group: number of mice for each organ differs, but n = at least 6 for each organ. **Panel C-D**) Pooled from 2 independent experiments for each group: control (n = 11 mice) and neutrophil depletion (n = 7 mice). **Panel E**) Pooled from at least 3 independent experiments for each group: number of mice for each day differs, but n = at least 8 for each day. **Panel F**) Pooled from at least 5 independent experiments for each group: number of mice for each organ differs, but n = at least 14 for each organ. Two-tailed Mann Whitney-U tests were used to compare two groups in each panel. P≥0.05 not significant (ns), p<0.05 (*), p<0.01 (**), p>0.0001 (****).
(TIF)

S2 Fig Consequences of PAD4 inhibition on luminal pathogen loads and systemic spread (related to Fig 3). **A)** Streptomycin pretreated C57BL/6 mice were infected orally with 5x10^7^ CFU of wild-type *S*.Tm for 3 days. One group (control from **S1A and S1F Fig**) treated with the vector (PBS; black symbols) and the second group with PAD4 inhibitor (GSK484; purple symbols) intraperitoneally (I.P.). *S*.Tm pathogen loads **A)** in feces until day 3 p.i. (CFU / g) and **B)** in systemic organs at day 3 p.i. (CFU (organ). **C)** Relative ranks of the tagged *S*.Tm strain abundances in feces at day 3 p.i.. **D)** Evenness score at day 3 p.i. Upper ends of the bars indicate the median. **Panels A-D**) Pooled from 2 independent experiments for each group: control (n = 11 mice) and PAD4 inhibition (n = 5 mice). Two-tailed Mann Whitney-U tests were used to compare two groups in each panel. p≥0.05 not significant (ns).
(TIF)

S3 Fig Analysis of fecal and systemic pathogen loads in a mouse model with reduced systemic disease (related to Fig 5). **A)** Experimental scheme for **Panels B-D**. Streptomycin or ampicillin pretreated C57BL/6 mice were infected orally with 5x10^7^ CFU of wild-type or SPI-2 mutant *S*.Tm (*S*.Tm^*ssaV*^) for 3 days. **B-C)** Total pathogen loads in cecal content in **B)** wt *S*.Tm infected and **C)** *S*.Tm^*ssaV*^ infected mice. **D)** Quantification of gut inflammation by Lipocalin-2 levels in cecal content of *S*.Tm^*ssaV*^-infected mice. **E-G)** Additional analysis of mice from **Fig 5A**. Total *S*.Tm^*ssaV*^ pathogen loads **E)** in feces at day 1–3, **F)** in systemic organs (mLN and spleen) at day 3 p.i. in each group. **G)** Quantification of mRNA expression levels in the cecal tissue by qRT-PCR. Results are represented relative to β-actin mRNA levels. Upper ends of the bars indicate the median. Strep. + wt *S*.Tm; n = 5, Amp. + wt *S*.Tm; n = 5, Strep. + *S*.Tm^*ssaV*^; n = 10, Amp. + *S*.Tm^*ssaV*^; n = 14 (12 replotted from **Fig 5B**). **Panels E-G-**) Pooled from total 4 independent experiments; at least 2 for each group: Group-1 (n = 12 mice), group-2 (n = 9 mice), group-3 (n = 6 mice), group-4 (n = 6 mice). Two-tailed Mann Whitney-U tests were used to compare two indicated groups in each panel. p≥0.05 not significant (ns), p<0.05 (*), p<0.01 (**), p<0.001 (***).
(TIF)

S4 Fig Microscopy analysis of cecal tissue in terms of its regeneration capacity (related to Fig 6). **A)** Representative micrographs of cecal tissue sections, stained for epithelial marker EpCam and cell division marker Ki67. LPS. Lu. = Lumen.. Ep. = Epithelium. L.P. = Lamina Propria. White arrows point at regions with gaps in the epithelial barrier. Scale bar = 50 μm.
(TIF)

S5 Fig Effect of neutrophil depletion on epithelial integrity in OligoMM12 mice with unperturbed microbiota (related to Fig 6). **A)** Experimental scheme. OligoMM^12^ mice were infected orally with 5x10^7^ CFU of wt *S*.Tm for 4 days. Treated with either PBS (n = 3) or α-Ly6G (n = 3). Total pathogen loads **B)** in cecal content, and **C)** in mLN. **D)** Quantification of mRNA expression levels in the cecal tissue by qRT-PCR. Results are represented relative to uninfected control. **E)** Representative micrographs of cecal tissue sections, stained for epithelial marker EpCam and *Salmonella* LPS. Lu. = Lumen. Ep. = Epithelium. White arrows point at expelled epithelial cells. Scale bar = 50 μm. **F**) Microscopy-based quantification of luminal IECs per 63x field of view (i.e., cells/high power field; hpf). Two-tailed Mann Whitney-U tests were used to compare two indicated groups in each panel. p≥0.05 not significant.
(TIF)

S6 Fig Proposed working model explaining the sequence of infection events in the presence and absence of intraluminal neutrophils. **A)** Presence of intraluminal neutrophil defense. Stage 1: *Salmonella* invasion of the epithelium is sensed by the NAIP/NLRC4 inflammasome in epithelial cells, resulting the expulsion of infected cells into the gut lumen [12]. This leads to shortening of the crypts. At the same time, inflammasome signalling promotes recruitment of immune cells, including neutrophils, into the lamina propria. Stage 2: Neutrophils swarm into the gut lumen where they attack the invading pathogen cells and form aggregates consisting of neutrophils, NETs, and *Salmonella*. Stage 3: This barrier formed by neutrophils block further *Salmonella* attacks on the epithelium temporarily, which allows epithelial progenitor cells enough time to divide and re-establish the barrier. **B)** Absence of intraluminal neutrophil defense. Stage 1: *Salmonella* invasion of the epithelium is sensed by the NAIP/NLRC4 inflammasome in epithelial cells, resulting the expulsion of infected cells into the gut lumen. This leads to shortening of the crypts. No neutrophils are recruited. Stage 2: This leaves the epithelium exposed to further *Salmonella* attacks as the luminal bacterial population is not faced with a neutrophil counterattack. As a result, the pathogen cells continue to assault the epithelium. Epithelial cells continue to expel in response to these attacks, eventually leading to uncontrolled epithelial cell loss. Stage 3: Massive and continuous shedding result in extremely short crypts and gap formation. The epithelial barrier is breached. Underlying tissue is in direct contact with the gut luminal content.
(TIF)
