# Supplementary figures and images for: Intraluminal neutrophils limit epithelium damage by reducing pathogen assault on intestinal epithelial cells during Salmonella gut infection
Source: PLoS Pathog. 2023 Jun 29;19(6):e1011235. doi: 10.1371/journal.ppat.1011235 (PMC10337893; doi:10.1371/journal.ppat.1011235)

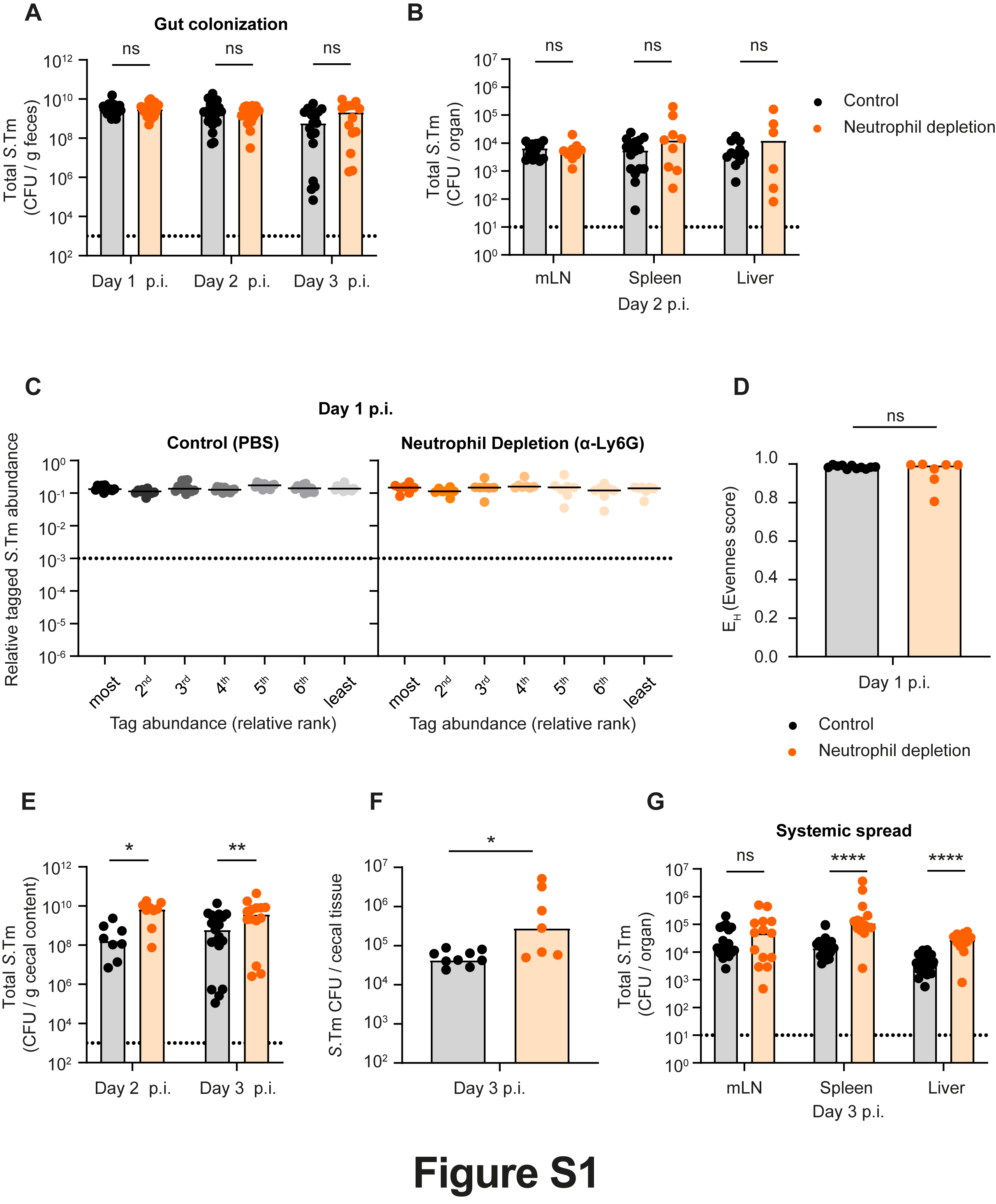

Supplement: S1 Fig — A) Streptomycin pretreated C57BL/6 mice were infected orally with 5x10^7^ CFU of wild-type S.Tm (SL1344) for 3 days. One group (control) was treated with the vector (PBS; black symbols) and the second group with α-Ly6G (orange symbols) intraperitoneally (I.P.). S.Tm pathogen loads A) in feces until day 3 p.i. (CFU / g) and B) in systemic organs at day 2 p.i. (CFU / organ). C) Relative ranks of the tagged S.Tm strain abundances in feces at day 1 p.i.. D) Evenness score at day 1 p.i. E-F) S.Tm pathogen loads E) in the cecal content at day 2 and 3 p.i. (CFU / g) and F) in systemic organs (mLN, spleen, and liver) at day 3 p.i. (CFU / organ). Lines or upper ends of the bars indicate the median. Dotted lines indicate the detection limit. G) S.Tm pathogen loads in cecal tissue (CFU / g). Panel A) Pooled from 9 independent experiments for each group: number of mice each day differs as experiments terminated at day 1, 2 or 3 p.i., but n = at least 14 at each day. Panel B) Pooled from 4 independent experiments for each group: number of mice for each organ differs, but n = at least 6 for each organ. Panel C-D) Pooled from 2 independent experiments for each group: control (n = 11 mice) and neutrophil depletion (n = 7 mice). Panel E) Pooled from at least 3 independent experiments for each group: number of mice for each day differs, but n = at least 8 for each day. Panel F) Pooled from at least 5 independent experiments for each group: number of mice for each organ differs, but n = at least 14 for each organ. Two-tailed Mann Whitney-U tests were used to compare two groups in each panel. P≥0.05 not significant (ns), p<0.05 (*), p<0.01 (**), p>0.0001 (****). (TIF) [file ppat.1011235.s001.tif]

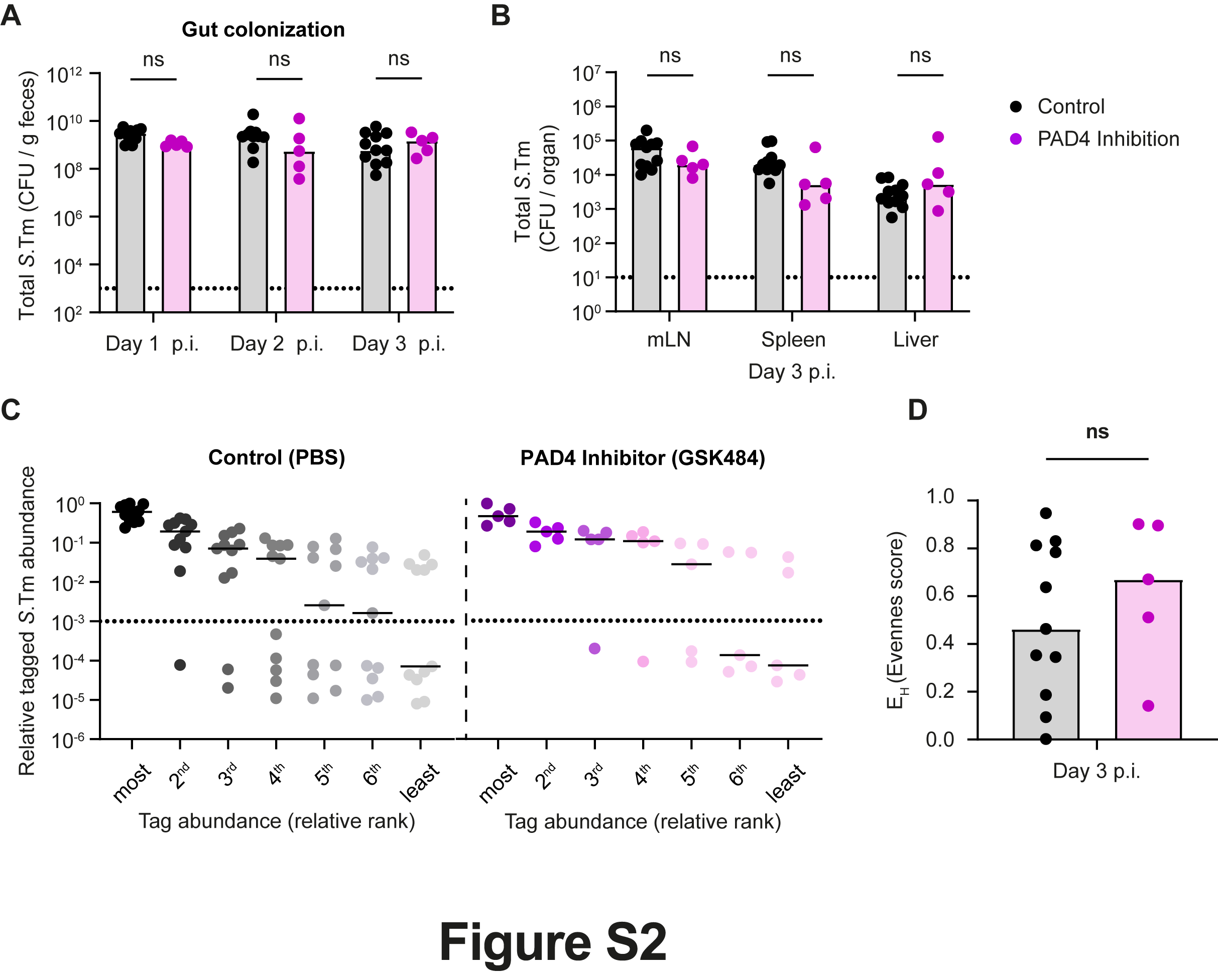

Supplement: S2 Fig — A) Streptomycin pretreated C57BL/6 mice were infected orally with 5x10^7^ CFU of wild-type S.Tm for 3 days. One group (control from S1A and S1F Fig) treated with the vector (PBS; black symbols) and the second group with PAD4 inhibitor (GSK484; purple symbols) intraperitoneally (I.P.). S.Tm pathogen loads A) in feces until day 3 p.i. (CFU / g) and B) in systemic organs at day 3 p.i. (CFU (organ). C) Relative ranks of the tagged S.Tm strain abundances in feces at day 3 p.i.. D) Evenness score at day 3 p.i. Upper ends of the bars indicate the median. Panels A-D) Pooled from 2 independent experiments for each group: control (n = 11 mice) and PAD4 inhibition (n = 5 mice). Two-tailed Mann Whitney-U tests were used to compare two groups in each panel. p≥0.05 not significant (ns). (TIF) [file ppat.1011235.s002.tif]

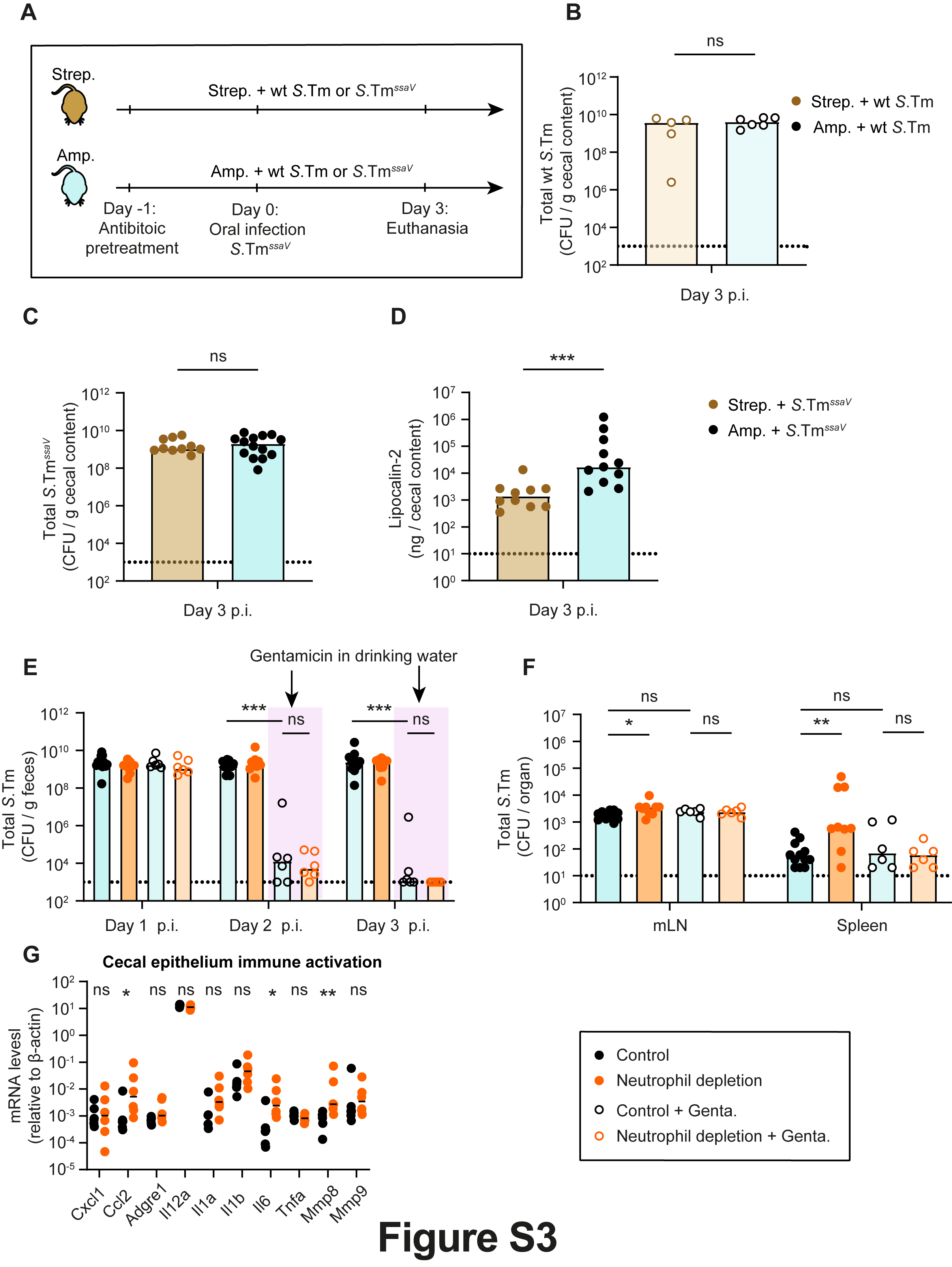

Supplement: S3 Fig — A) Experimental scheme for Panels B-D. Streptomycin or ampicillin pretreated C57BL/6 mice were infected orally with 5x10^7^ CFU of wild-type or SPI-2 mutant S.Tm (S.TmssaV) for 3 days. B-C) Total pathogen loads in cecal content in B) wt S.Tm infected and C) S.TmssaV infected mice. D) Quantification of gut inflammation by Lipocalin-2 levels in cecal content of S.TmssaV-infected mice. E-G) Additional analysis of mice from Fig 5A. Total S.TmssaV pathogen loads E) in feces at day 1–3, F) in systemic organs (mLN and spleen) at day 3 p.i. in each group. G) Quantification of mRNA expression levels in the cecal tissue by qRT-PCR. Results are represented relative to β-actin mRNA levels. Upper ends of the bars indicate the median. Strep. + wt S.Tm; n = 5, Amp. + wt S.Tm; n = 5, Strep. + S.TmssaV; n = 10, Amp. + S.TmssaV; n = 14 (12 replotted from Fig 5B). Panels E-G-) Pooled from total 4 independent experiments; at least 2 for each group: Group-1 (n = 12 mice), group-2 (n = 9 mice), group-3 (n = 6 mice), group-4 (n = 6 mice). Two-tailed Mann Whitney-U tests were used to compare two indicated groups in each panel. p≥0.05 not significant (ns), p<0.05 (*), p<0.01 (**), p<0.001 (***). (TIF) [file ppat.1011235.s003.tif]

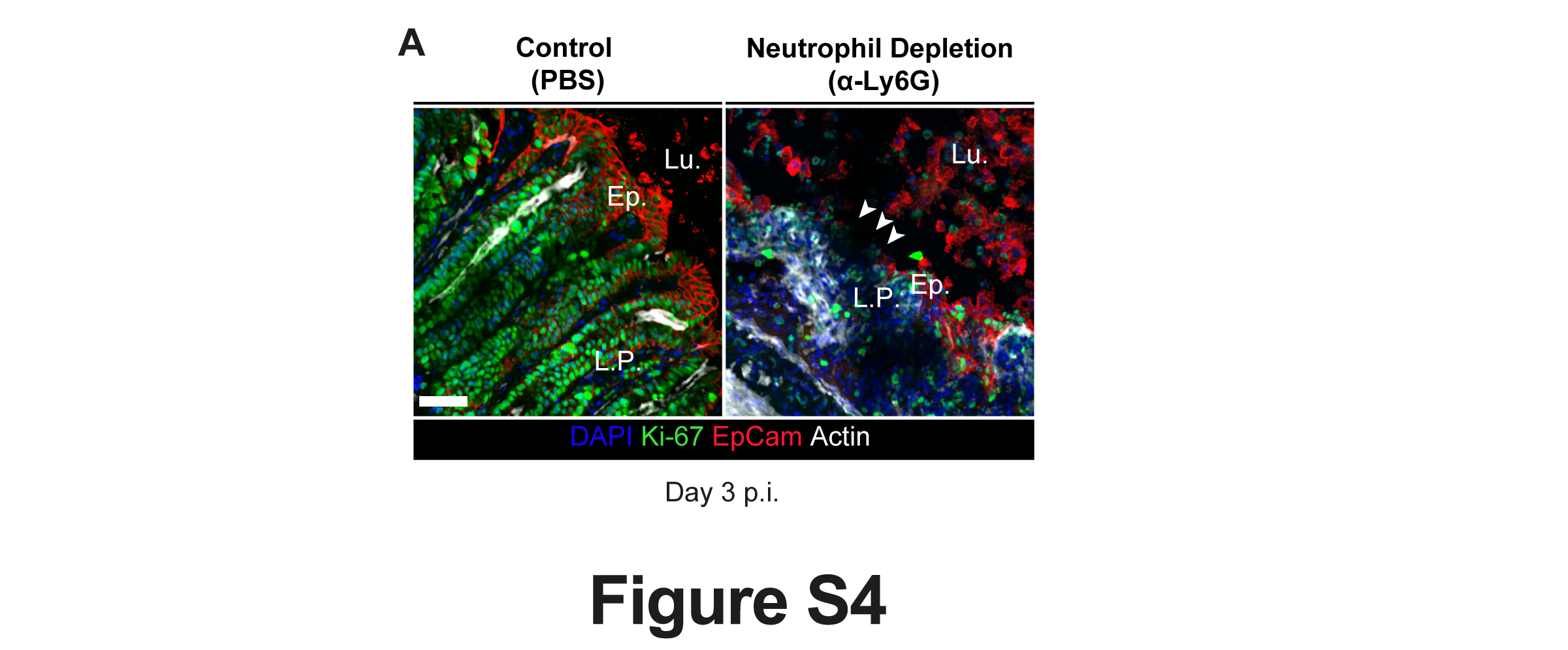

Supplement: S4 Fig — A) Representative micrographs of cecal tissue sections, stained for epithelial marker EpCam and cell division marker Ki67. LPS. Lu. = Lumen.. Ep. = Epithelium. L.P. = Lamina Propria. White arrows point at regions with gaps in the epithelial barrier. Scale bar = 50 μm. (TIF) [file ppat.1011235.s004.tif]

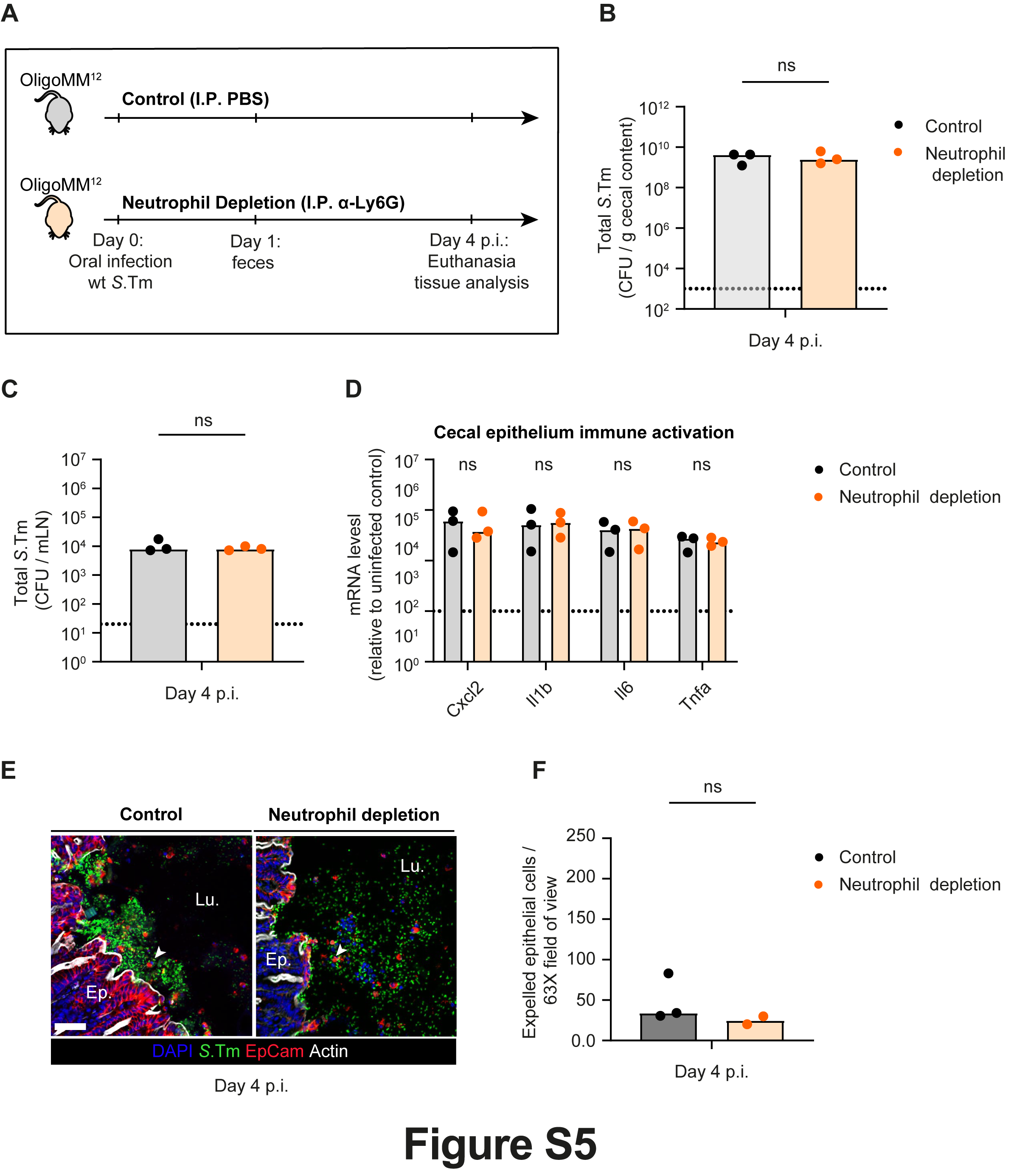

Supplement: S5 Fig — A) Experimental scheme. OligoMM12 mice were infected orally with 5x10^7^ CFU of wt S.Tm for 4 days. Treated with either PBS (n = 3) or α-Ly6G (n = 3). Total pathogen loads B) in cecal content, and C) in mLN. D) Quantification of mRNA expression levels in the cecal tissue by qRT-PCR. Results are represented relative to uninfected control. E) Representative micrographs of cecal tissue sections, stained for epithelial marker EpCam and Salmonella LPS. Lu. = Lumen. Ep. = Epithelium. White arrows point at expelled epithelial cells. Scale bar = 50 μm. F) Microscopy-based quantification of luminal IECs per 63x field of view (i.e., cells/high power field; hpf). Two-tailed Mann Whitney-U tests were used to compare two indicated groups in each panel. p≥0.05 not significant. (TIF) [file ppat.1011235.s005.tif]

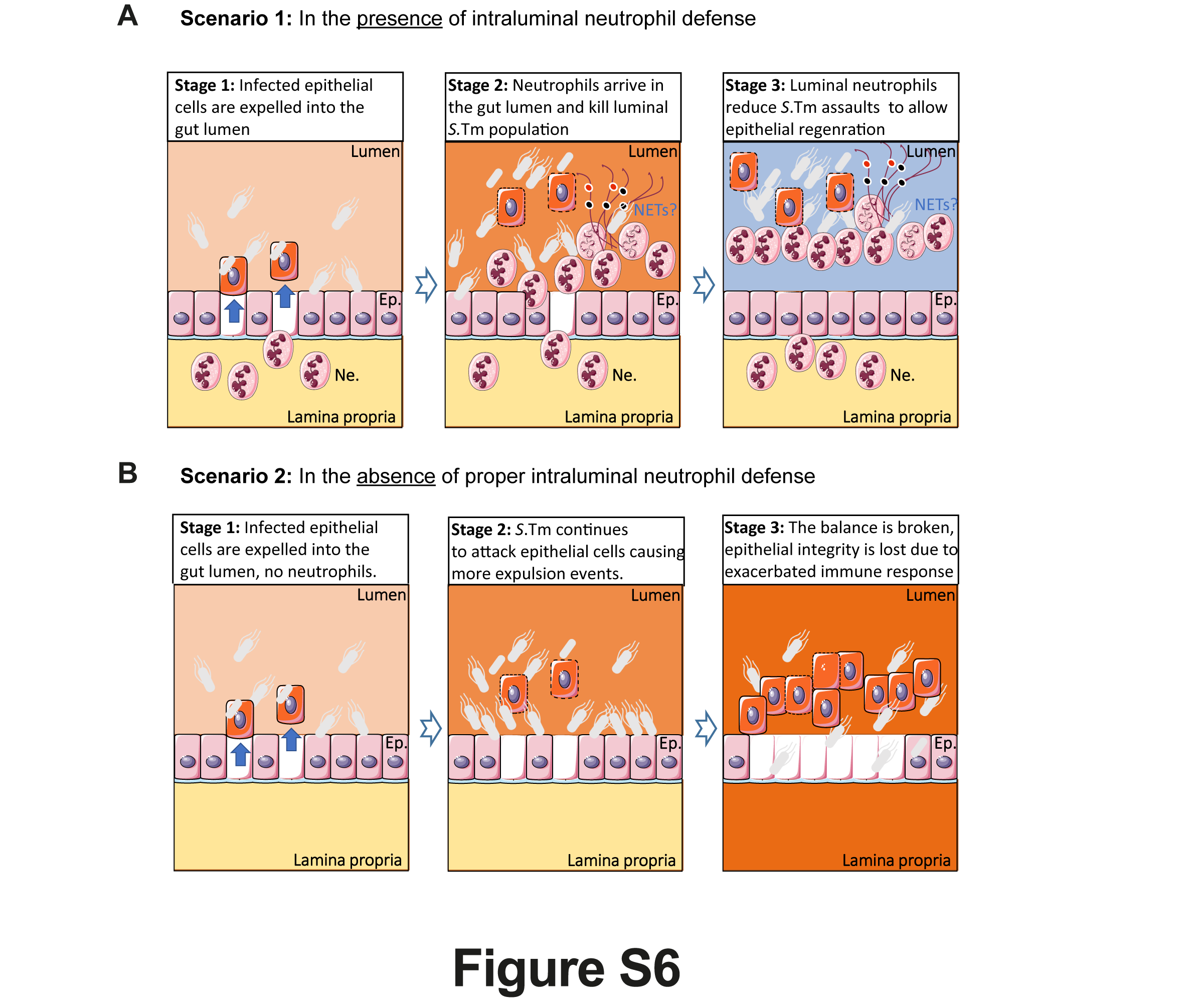

Supplement: S6 Fig — A) Presence of intraluminal neutrophil defense. Stage 1: Salmonella invasion of the epithelium is sensed by the NAIP/NLRC4 inflammasome in epithelial cells, resulting the expulsion of infected cells into the gut lumen [12]. This leads to shortening of the crypts. At the same time, inflammasome signalling promotes recruitment of immune cells, including neutrophils, into the lamina propria. Stage 2: Neutrophils swarm into the gut lumen where they attack the invading pathogen cells and form aggregates consisting of neutrophils, NETs, and Salmonella. Stage 3: This barrier formed by neutrophils block further Salmonella attacks on the epithelium temporarily, which allows epithelial progenitor cells enough time to divide and re-establish the barrier. B) Absence of intraluminal neutrophil defense. Stage 1: Salmonella invasion of the epithelium is sensed by the NAIP/NLRC4 inflammasome in epithelial cells, resulting the expulsion of infected cells into the gut lumen. This leads to shortening of the crypts. No neutrophils are recruited. Stage 2: This leaves the epithelium exposed to further Salmonella attacks as the luminal bacterial population is not faced with a neutrophil counterattack. As a result, the pathogen cells continue to assault the epithelium. Epithelial cells continue to expel in response to these attacks, eventually leading to uncontrolled epithelial cell loss. Stage 3: Massive and continuous shedding result in extremely short crypts and gap formation. The epithelial barrier is breached. Underlying tissue is in direct contact with the gut luminal content. (TIF) [file ppat.1011235.s006.tif]
